# How Structured Is the Entangled Bank? The Surprisingly Simple Organization of Multiplex Ecological Networks Leads to Increased Persistence and Resilience

**DOI:** 10.1371/journal.pbio.1002527

**Published:** 2016-08-03

**Authors:** Sonia Kéfi, Vincent Miele, Evie A. Wieters, Sergio A. Navarrete, Eric L. Berlow

**Affiliations:** 1 Institut des Sciences de l’Evolution de Montpellier, BioDICée team, Université de Montpellier, CNRS, IRD, EPHE, CC 065, Montpellier, France; 2 Laboratoire Biométrie et Biologie Evolutive, CNRS, UMR5558, Université de Lyon, Villeurbanne, France; 3 Estación Costera de Investigaciones Marinas (ECIM), Center for Marine Conservation, LINC-Global, Chile; 4 Center for Applied Ecology and Sustainability (CAPES), Departamento de Ecología, Pontificia Universidad Católica de Chile, Santiago, Chile; 5 Vibrant Data Inc., San Francisco, California, United States of America; University of Tennessee, UNITED STATES

## Abstract

Species are linked to each other by a myriad of positive and negative interactions. This complex spectrum of interactions constitutes a network of links that mediates ecological communities’ response to perturbations, such as exploitation and climate change. In the last decades, there have been great advances in the study of intricate ecological networks. We have, nonetheless, lacked both the data and the tools to more rigorously understand the patterning of multiple interaction types between species (i.e., “multiplex networks”), as well as their consequences for community dynamics. Using network statistical modeling applied to a comprehensive ecological network, which includes trophic and diverse non-trophic links, we provide a first glimpse at what the full “entangled bank” of species looks like. The community exhibits clear multidimensional structure, which is taxonomically coherent and broadly predictable from species traits. Moreover, dynamic simulations suggest that this non-random patterning of how diverse non-trophic interactions map onto the food web could allow for higher species persistence and higher total biomass than expected by chance and tends to promote a higher robustness to extinctions.

## Abstract 2

An ecological network synthesizing all known interactions between species exhibits a clear pattern of organization that reflects evolutionary and ecological constraints operating in this entangled bank of species.

## Author Summary

Within an ecosystem, species interact with each other in many different ways, including predation, competition, and facilitation, and this can be modelled as a network of multiple interaction types. The variety of interaction types that link species to each other has long been recognized but has rarely been synthesized for entire multi-species ecosystems. Here, we leverage a unique marine ecological network that integrates thousands of trophic and non-trophic interactions. We show that, despite its multidimensional complexity, this ecological network collapses into a small set of “functional groups,” i.e., groups of species that resemble each other in the way they interact with others in their combined trophic and non-trophic interactions. These groups are taxonomically coherent and predictable by species attributes. Moreover, dynamic simulations suggest that the way the different interaction types relate to each other allows for higher species persistence and higher total biomass than is expected by chance alone, and that this tends to promote a higher robustness to extinctions. Our results will help to guide future empirical studies and to develop a more general theory of the dynamics of complex ecological systems.

## Introduction

In his description of the “entangled bank” of species, Darwin illustrated the principle that species must manage complex interdependencies to successfully coexist in natural communities [1–7]. In this context, evolutionary constraints set a landscape of trade-offs over which species must solve their basic needs within the context of other species (e.g., competition for refuges among herbivores forced by the common need to avoid predators) and stringent environmental conditions. To some extent, each species has found unique solutions—in how they manage interactions with other species—that have shaped their distinctive niches.

However, beyond species identity, common sets of trade-offs may lead to similarities in the way species are involved in different interaction types. In other words, the apparently endless solutions discovered by species to simultaneously satisfy multiple requirements and deal with multiple stresses might actually be much more limited and structured than we anticipated. Yet we do not know what the full “entangled bank” of species looks like or if there are structural patterns at the community level that reflect common solutions in the way species manage being involved in different interaction types. Indeed, the analysis tools from network science are only recently addressing the “multiplex” nature of most natural networks, i.e., the fact that they include different interaction types between a given set of species (e.g., [8–11]).

As the first datasets including several interaction types between a given set of species are now emerging in ecology [5,12–16], we have a unique opportunity to disentangle the bank of species interactions. Until now, layers in such ecological networks have been analyzed separately from each other; i.e., the structure of trophic webs has been analyzed independently of the structure of competition or mutualistic webs ([13–15,17–19], but see [5]). However, the way network layers are intertwined with each other matters for community dynamics and resilience [1,2,20]. Thus, it is critical to move beyond unidimensional analyses of ecological networks.

In this paper, we explore a comprehensive ecological network in which the species of a local community are linked by trophic and widely diverse positive and negative non-trophic interactions [14,21]. The network, hereafter referred to as the Chilean web, includes three layers of interactions among 106 co-occurring species in the marine rocky intertidal community of the central coast of Chile: a trophic layer (i.e., a food web; 1,362 trophic links), a negative non-trophic layer (e.g., interference, competition for space; 3,089 links), and a positive non-trophic layer (e.g., habitat/refuge provisioning by sessile species that create structure for others; 172 links), making it a three-dimensional multiplex network [9,11]. We first quantified the three-dimensional structure of this multiplex network using a probabilistic clustering method. We then used dynamical modeling to investigate how the identified structure modulates the multi-species dynamics and the resilience of the ecological community to perturbations. Overall, our results suggest that the enormous ecological complexity of this community can be simplified into surprisingly clear patterns of organization that are taxonomically coherent, can be broadly predicted from simple species traits, and are functionally important for dynamics and resilience. These blocks might represent ecological and evolutionary constraints acting on the multiple requirements and impacts that allow species to persist in complex systems. Our results, therefore, pave the way for a new generation of research untangling complex networks with multiple link types.

## Results

### The Multiplex Pairwise Interactions

Looking at the way pairs of species are three-dimensionally connected in the Chilean web shows that 2,891 of these pairwise links are interaction-specific (Table 1; S1 Fig). In other words, pairs of species tend to engage in only one type of interaction: trophic, positive non-trophic, or negative non-trophic interactions. We compared these occurrences to those observed in random multiplex networks with the same expected degree sequence as in the Chilean web (see Materials and Methods). Note that these random networks are very constrained and are, as a consequence, very similar to the Chilean web (S9 and S10 Figs). We found that the interaction-specific links (i.e., the cases in which a pair of species is linked by only one interaction type) are significantly more frequent in the Chilean web than expected in the random counterparts (*p*-value < 10^−4^; Table 1). In contrast, 125 pairs involve two interaction types simultaneously, which is far less than expected (*p*-value < 10^−4^; Table 1). Notably, six pairs of species are linked at the same time by the three interaction types in this interaction web, which is more than expected (*p*-value < 10^−2^; Table 1). These patterns suggest a fine-scale, species-level constraint on how pairs of species interact in webs with several interaction types; i.e., multiplex pairwise interactions are remarkably rare. It does not mean that species are not involved in multiple interaction types; they usually are, but with different partners.

**Table 1:** Pairwise interactions observed in the Chilean web compared to the minimum and maximum values observed in random multiplex networks simulated layer by layer.

	Observed	Random Range	*P*-value
**One interaction type**	2,891	2,705–2,884	<10^−5^
**Two interaction types**	125	154–228	<10^−5^
**All interaction types**	6	0–9	0.0094

Underlying data can be found in the Dryad repository: http://dx.doi.org/10.5061/dryad.b4vg0 [21].

This lack of multiplex pairwise interactions may reflect evolutionary constraints in developing adaptations simultaneously for different interaction types with the same species. For example, in the Chilean web, it is relatively rare for a species to facilitate its prey (there are only two pairs of species simultaneously linked by a trophic and a facilitation link). One exception is the scurrinid limpet *Scurria variabilis*, which lives on top of the shell of another limpet, the keyhole limpet *Fissurela limbata*, which, in turn, can eat the juveniles of *S*. *variabilis* [22]. The positive effect on *S*. *variabilis* is quite strong, since they can spend their entire benthic life grazing on the *Fissurella* shells [22,23]. However, it is likely that the trophic link is weak, because the species are primarily herbivores [24–26], which would reinforce the notion that such combination of interaction types is rare. There are, however, more examples in the Chilean web of species that compete with their prey or with their predator (e.g., anemones eat mussels and compete for space with them), of species facilitating their competitor (e.g., algae facilitate mussel recruitment but compete for space once mussels are established) [27], and, interestingly, of prey facilitating their own predators (e.g., mussels facilitate settlement of their predatory crabs) [4]. While these types of examples tend to dominate our intuitive perception of insurmountable ecological complexity, the data suggests that they are the exception, not the rule.

### The Multiplex Clusters

When we take into account all three types of interactions, as well as the identity of the participants, do groups of species have similar interaction profiles? To address that question, we used a probabilistic algorithm to detect groups of species (hereafter referred to as “multiplex clusters”) that resemble each other in the way they interact with others in their combined trophic and non-trophic interactions (i.e., the way they interact in three dimensions). Our work hereby builds on previous efforts aimed at detecting compartments [28,29] or structural patterns [30] in food webs but extends those approaches to networks with several interaction types. In particular, previous studies have used similar approaches to characterize the trophic niche of species by identifying “trophic species”, i.e., groups of species that are similar in terms of their predators and prey. Here, our approach applied to the Chilean web allows, for the first time, to our knowledge, the visualization of the multidimensional ecological niche of species [31].

**Fig 1:**
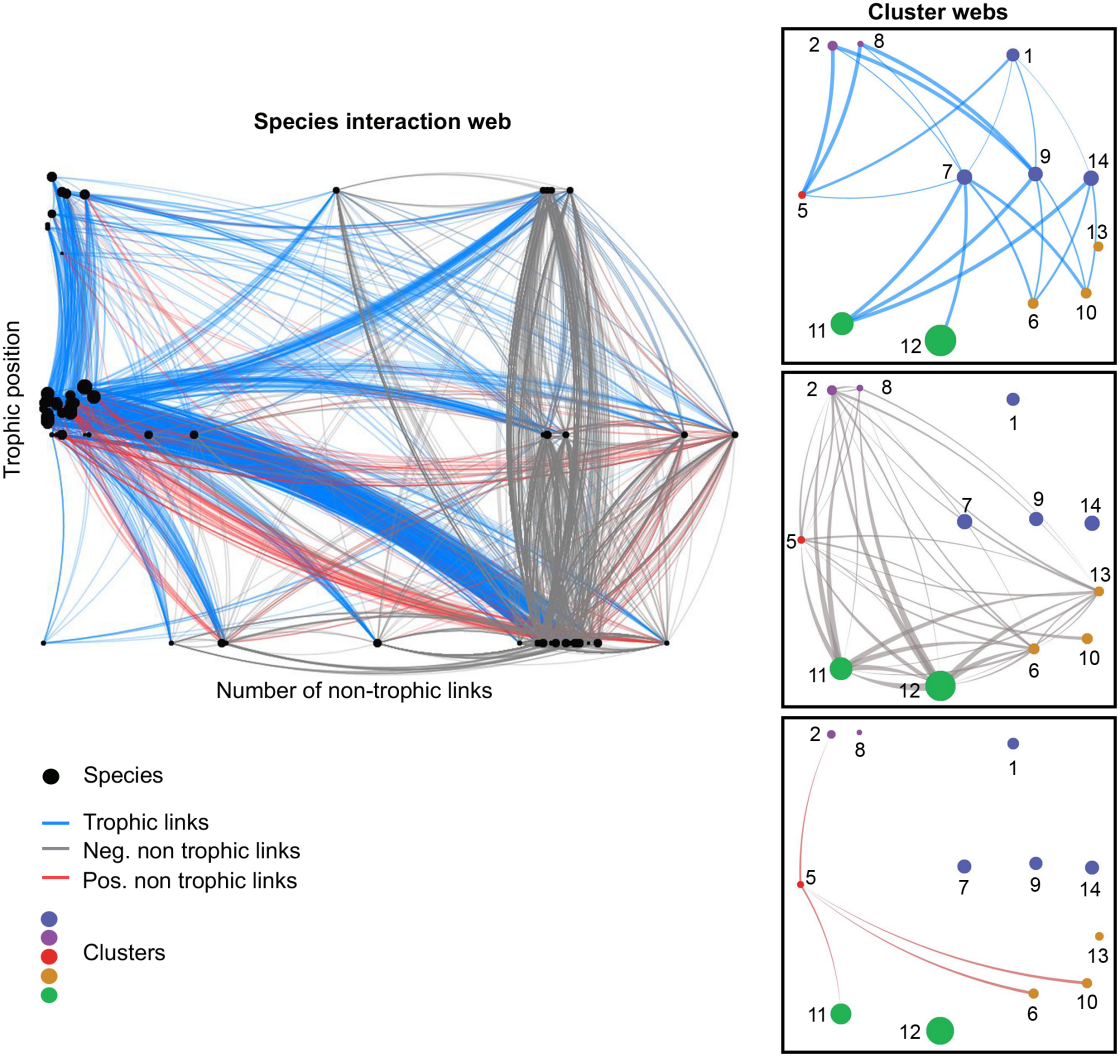
From species to multiplex clusters. **Left:** Network of trophic and non-trophic interactions between the 106 species of the Chilean web. Nodes indicate species and are sized by total degree. Vertical position is proportional to trophic level. Horizontal position is proportional to non-trophic degree. Edges are blue, red, and gray for trophic, positive, and negative interactions, respectively. Edges’ directionality is represented by link curvature, with lines arching clockwise from source to target. **Right:** Interactions between the multiplex clusters. Nodes are sized by the number of species in the cluster. Numbers correspond to the cluster ID used in the text. Link widths are proportional to the interaction probability between clusters. Only edges whose probability is superior to 0.5 are plotted, and cluster 3 (benthic diatoms) is not shown. Cluster 4 is absent because it is not involved in any interaction type with a probability >50%. Clusters of the same color have similar 3D connectivity but differ in the identity of interacting species. These colors reflect the “multiplex functional groups” defined later on. The networks were plotted with VibrantData (http://vibrantdata.io). Underlying data can be found in the Dryad repository: http://dx.doi.org/10.5061/dryad.b4vg0 [21].

**Fig 2:**
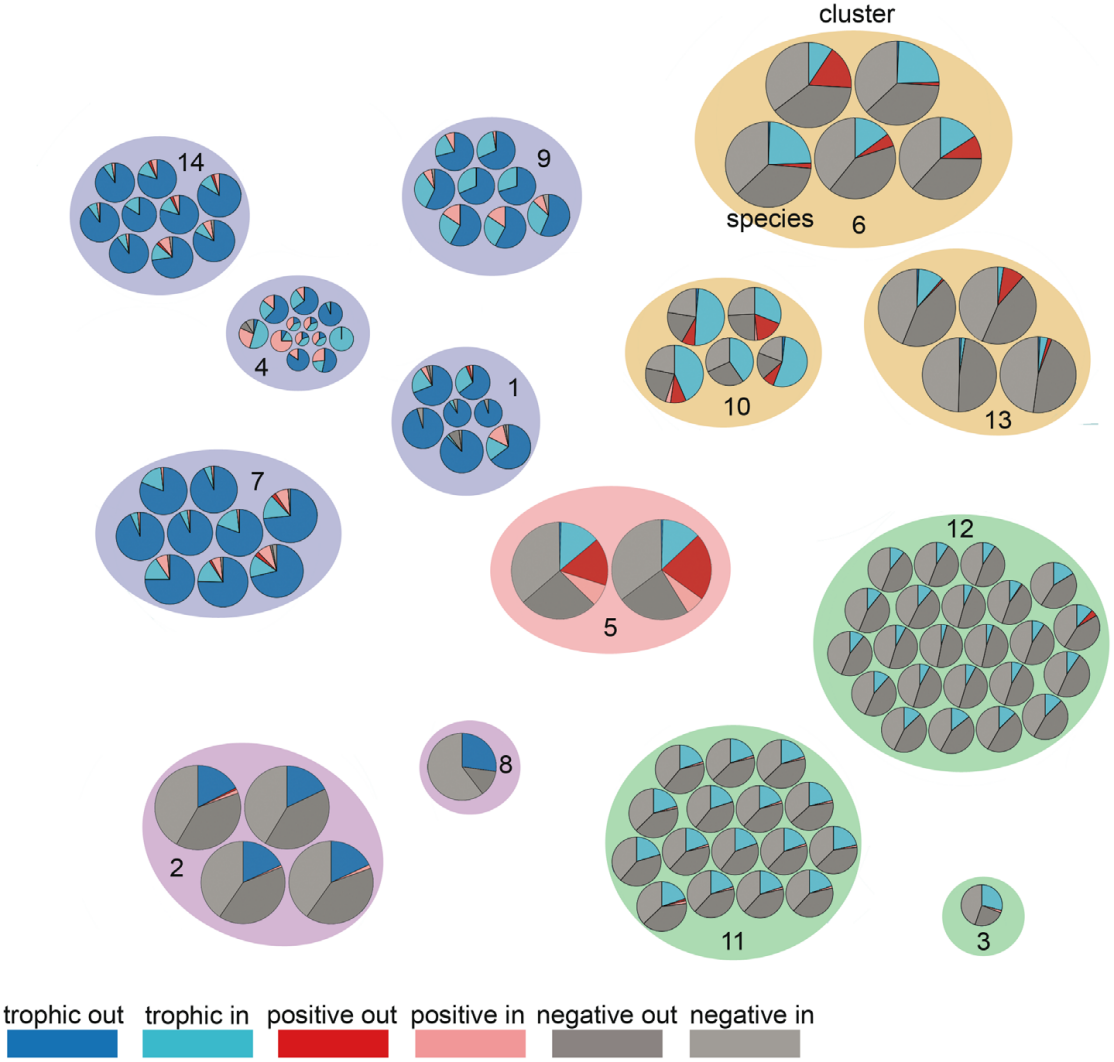
Species’ 3D incoming and outgoing degrees. Pies represent the relative involvement of the 106 species in trophic (blue), negative (grey), and positive (red) non-trophic interactions; darker (resp. lighter) color represents outgoing (resp. incoming) links (legend on the bottom left). Pie diameter is proportional to the species total degree. Ellipses around groups of species represent the multiplex clusters. Numbers correspond to the cluster ID used in the main text. Clusters of the same color have similar 3D connectivity but differ in the identity of interacting species; i.e., they belong to the same “multiplex functional group” defined later on. Please see http://pbil.univ-lyon1.fr/software/multiplex for an interactive version of the figure. Underlying data can be found in the Dryad repository: http://dx.doi.org/10.5061/dryad.b4vg0 [21].

When applied to the Chilean web, and associated with a model selection procedure, the probabilistic algorithm identified 14 multiplex clusters, i.e., much less than the number of species (Figs 1 and S2). Those clusters differ from each other in the types of links they are involved in, the pattern of incoming and outgoing links (Fig 2), and the identity of the species they interact with (S4 and S5 Figs). We note that the definition of the clusters requires taking into account the three layers of interactions simultaneously, because none of the layers contains by itself enough information to recover these multiplex clusters (S6 Fig, S1 Table and S1 Text).

Clusters 2, 5, and 8 are the cornerstone of that organization, both because of the high frequency of interactions engaged in with others and because of the variety of their interaction partners (Figs 1 and 2). Cluster 5 is an overall hub of interactions, with both a high frequency and a wide variety of interactions with others (Figs 1 and 2). Clusters 6 and 10 are two groups of species involved in similar interaction types and partners but that do not have a single interaction with each other (S4 and S5 Figs); indeed, the two groups of species are spatially segregated across the tidal gradient, with one group typically found in the lower shore (cluster 6) and the other found at the uppermost level (cluster 10). Most of the remaining clusters contain more species (7 to 23 species) that are, from a connectivity point of view, redundant and exchangeable. These clusters differ from one another by the identity of the species they interact with (e.g., clusters 9 and 7 are more generalist consumers than cluster 14), but also by the way they interact with the species of clusters 2, 5, and 8 (e.g., cluster 11 is facilitated while 12 competes with cluster 5; S4 and S5 Figs). In particular, cluster 4 comprises peripheral species that share a low interacting frequency with the other clusters.

The cluster number and their species composition was largely conserved after removal of up to 30% of the species in the Chilean web (S3 Fig and S1 Text). This shows that the probabilistic algorithm is robust against perturbations due to species removal but also that the retrieved organization is significant. This is, however, not unexpected since, in essence, the multiplex clusters gather species that share similar interaction patterns and are therefore largely substitutable in terms of their multiplex connectivity.

### Dynamical Consequences of the Non-trophic Interactions

Do the specific combinations of trophic and non-trophic links characterizing the clusters have functional consequences? We examined the relationship between the multiplex connectivity pattern identified in the Chilean web and the dynamic behavior of this network. To this end, we used a bio-energetic consumer-resource model (as in [32]) in which we incorporated the broad categories of non-trophic interactions found in the Chilean web. Because of species redundancy in the interaction patterns within a cluster, in this initial investigation, we used the clusters as the simulation units of the model. Later refinements should relax this assumption and look into the coherence of species dynamics within clusters. We compared the dynamics of (i) the webs of the 14 clusters identified in the Chilean web to (ii) equivalent random webs in which all non-trophic links were randomized throughout the web (see Materials and Methods).

**Fig 3:**
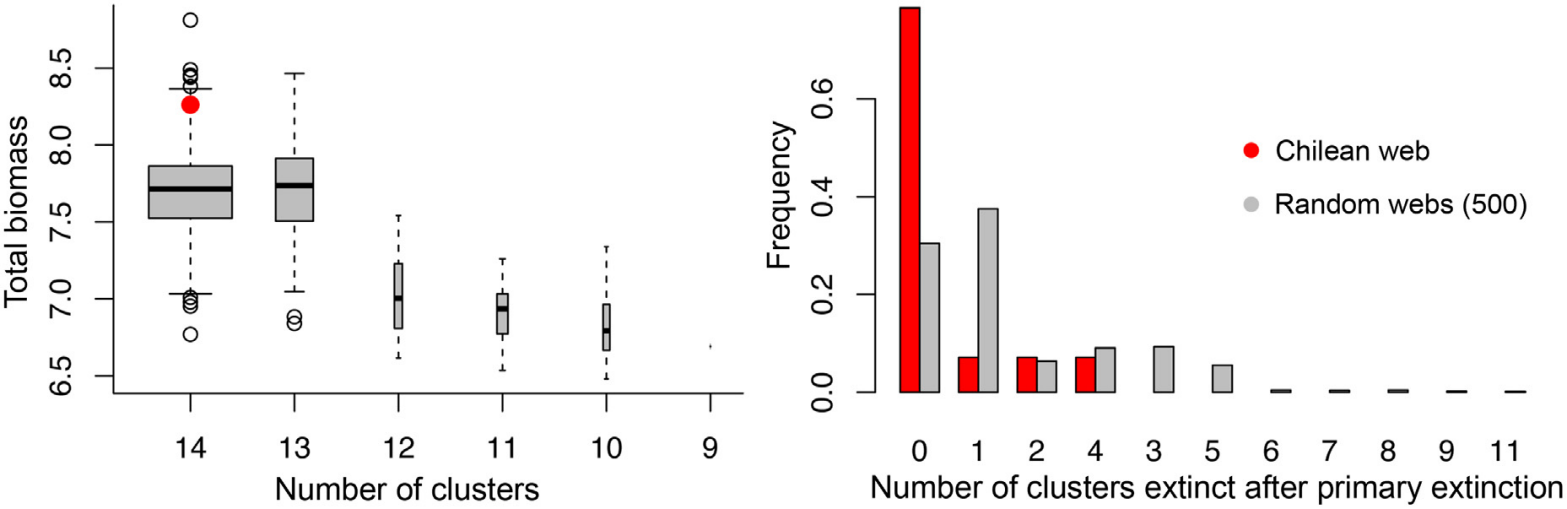
Example of the effect of the structure of non-trophic interactions on network dynamics. Dynamics of the 14 clusters were run in cases in which the three-dimensional interaction pattern was either the one of the Chilean web (red) or of 500 random networks (grey). In these random networks, the trophic layer is kept constant but the non-trophic links are randomized. See S2 Table for details on parameter values and S8 Fig for a discussion on the sensitivity of the results. **Left:** Box plot of the final biomass in the 500 random webs as a function of the number of remaining clusters at the end of the simulations. Box width is log-proportional to the counts. Red dot is the position of the configuration observed in the Chilean web (significant biomass difference, *p*-value = 0.028). **Right:** Distribution of the number of extinct clusters after the removal of one cluster in the Chilean web (red) and in the 500 random networks (grey), i.e., the number of secondary extinctions. The difference between the two distributions (red and grey) is visible but not statistically significant (chi-square, *p*-value = 0.0879). Underlying data can be found in the Dryad repository: http://dx.doi.org/10.5061/dryad.b4vg0 [21].

Simulation results suggest that the way non-trophic interactions are mapped onto the trophic ones in the Chilean web tends to increase species persistence and the total biomass realized (Fig 3 left), as compared to a random allocation of non-trophic interactions. This occurs for a broad range of trophic and non-trophic parameter values (S8 Fig and S1 Text). Moreover, the mapping of the non-trophic interactions in the Chilean web tends to decrease secondary extinctions (Fig 3 right). The different clusters had very different effects on web dynamics. For instance, biomass loss was observed after the removal of the cornerstone clusters (clusters 2, 5, and 8) and at a higher level than expected (cluster 5, *p*-value = 0.056; clusters 2+8 jointly, *p*-value = 0.06; see S7 Fig).

### The Multiplex Functional Groups

**Fig 4:**
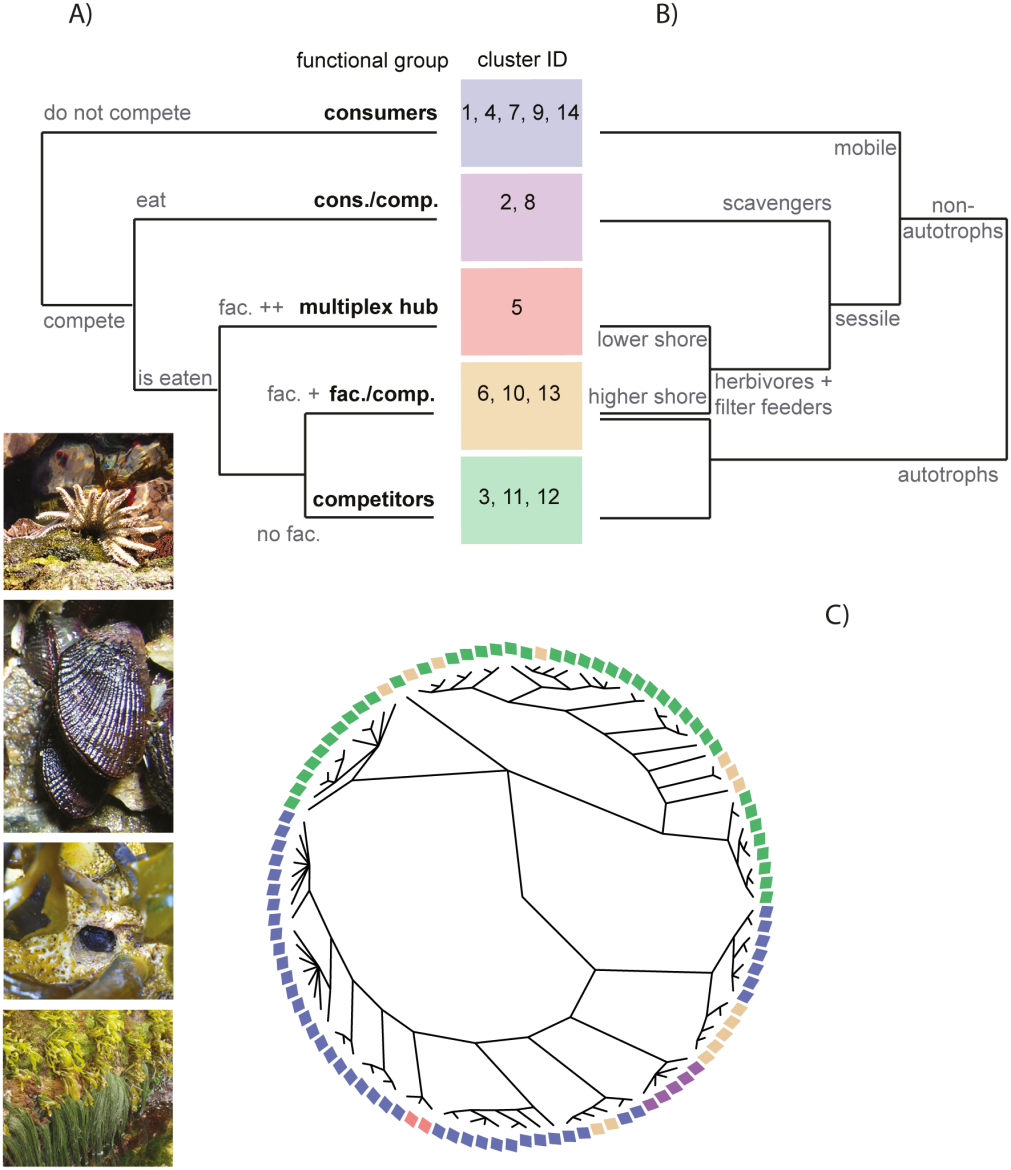
From species to multiplex functional groups. (A) and (B) Trees explaining the multiplex functional groups based on the species connectivity (B; see cluster dendogram, S11 Fig) and on species traits (C; see regression tree, S12 Fig). Rectangles represent the multiplex functional groups. Numbers correspond to the cluster ID used in the main text. (C) Species taxonomy with species colored by functional group (same colors as in Fig 2). The *p*-values of the different functional groups are: consumers (clusters 1, 4, 7, 9, 14): *p* < 1e-5; competitors (clusters 3, 11, 12): *p* = 1e-4; facilitators/competitors (clusters 6, 10, 13): *p* = 0.04 (not significant); consumers/competitors (anemones; clusters 2 and 8): *p* < 1e-5; multiplex hub (mussels; cluster 5): *p* < 1e-5. Pictures on the bottom left represent, from top to bottom, the predatory sea star *Heliaster helianthus* (cluster 1), the competitively dominant mussel *Perumytilus purpuratus* (cluster 5), the predatory crab *Acanthocyclus gayi* sheltering within the habitat-providing kelp *Lessonia spicata* (cluster 6), and a mixed assemblage of diverse algae species (picture credits: E. A. Wieters). Underlying data can be found in the Dryad repository: http://dx.doi.org/10.5061/dryad.b4vg0 [21].

If we go one step further and disregard the identity of the species, can we identify deeper cores of multiplex organization? By analyzing the interaction parameters estimated in the probabilistic model for the different clusters, we were able to identify groups of clusters whose species are involved (or not involved) in similar combinations of interactions, i.e., “multiplex functional groups” (Figs 4A and S11). The Chilean web thereby further collapses into a set of only five multiplex functional groups (Figs 4A and S11). Those multiplex functional groups can broadly be characterized as groups dominated by consumers (1, 4, 7, 9, 14), one composed mostly of competitors (3, 11, 12), another dominated by facilitators/competitors (6, 10, 13), a more heterogeneous group composed of consumers/competitors (2, 8), and, finally, one overall hub of species interacting with many other species in many different ways (5).

We find that the species composition of the functional groups is coherent with broad taxonomic classifications, considered as a coarse proxy for phylogenetic relatedness (Fig 4C). Each functional group has indeed a tendency to gather closely related species (*p*-value < 10^−4^). But exceptions exist. For instance, the group of facilitators/competitors (made of clusters 6, 10, 13) is composed of very different species corresponding to different phyla (mainly algae and barnacles; *p*-value > 0.1), but they share the fact that they are sessile species that create biotic structure for others.

Interestingly, the multiplex functional groups are not only characterized by similar multidimensional interaction pattern (by definition; Figs 4A and S11), but they are also very well predicted by simple species attributes (Figs 4B and S12), in particular trophic level category (autotroph, herbivore, intermediate, top), mobility (mobile versus sessile), and shore height (ordinal). The analysis first splits the data among autotroph species (mainly the competitors’ group and a few of the facilitators/competitors’ group) and the rest of the species. The second split separates mobile (the consumers’ group) from sessile species, which are then divided between carnivores (the consumers/competitors’ group) and herbivores, themselves split among species from lower (the multiplex hub and a few consumers) and those from higher shore (the facilitators/competitors’ group). Higher on the shore is more environmentally stressful because of increased exposure to air and desiccation [33,34]. It might, therefore, be more likely for sessile species at mid-high shore to facilitate mobile species that need shelter from environmental stress [35,36], while species lower on the shore are perhaps more likely to provide refuge from predation. Shore height could thereby mediate the frequency of facilitation of mobile by sessile species in this dataset.

In sum, the five multiplex functional groups gather species that engage in roughly similar ecological interactions (Fig 4): (1) A group of mobile consumers (clusters 1, 4, 7, 9, 14), mostly carnivores, composed of crabs, sea snails, chitons, starfishes, and birds, most of which consume prey species and often find themselves in competition with others. (2) A small group of sessile, inedible consumers (anemones; clusters 2 and 8) that eat dead or detached animals or their fragments are the source and target of many competitive links with other sessile species and are key players in the resilience of the community. Their classification into a separate group likely reflects their peculiar life habits (sessile scavengers). (3) An overall hub of sessile, edible consumers that also facilitate others and are key in the resilience of the community (cluster 5). This group contains two common mussel species that differentiate themselves from the other groups by their involvement in all interaction types and particularly in positive interactions (both incoming and outgoing; Figs 2, S4 and S5), supporting many ecological studies that highlight their role as foundational or engineering species [4,37,38]. They indeed provide habitat and substrate for many other invertebrate species seeking shelter. (4) A group of sessile primary producers (algae; clusters 3, 11, 12) that compete for space and usually find themselves in competitive loops while being frequently consumed. (5) Finally, a group of sessile species (clusters 6, 10, 13) that is a mix of algae and barnacles that compete for space with other sessile species while facilitating mobile consumers by creating biotic structure that provides refuges and habitat for other species (for instance, the kelp *Lessonia nigrescens* facilitates recruitment and provides critical shelter or habitat to diverse species).

## Discussion

The wave-exposed Chilean marine intertidal ecosystem of 106 species includes over 4,600 interactions that span predation, competition, and facilitation. Despite the wide range of possible combinations of interactions among species, our data suggests that the combinations of interactions that are actually realized in this intertidal community are constrained to be far fewer than those “possible.” Our analysis of the Chilean web further reveals a clear organization of species into a small subset of multiplex clusters, which themselves collapse into multiplex functional groups. The identification of this organization into clusters and, therefore, into functional groups requires taking into account the three layers of interactions and would not be possible with a monolayer, unidimensional niche approach of this ecological network.

The functional groups identified are taxonomically coherent, with each group gathering closely related species, suggesting some level of conservatism of the three-dimensional interaction niche space. The functional groups are also well-predicted by simple traits, such as trophic level, mobility, and shore height. Previous work on different single-interaction-type networks (food webs, bipartite mutualistic, and bipartite antagonistic) showed that only a limited number of traits is required to explain all species interactions in a given ecological network, meaning that ecological networks are structured by a few dimensions (or trait-axes) [31]. Our analysis of the Chilean web suggests that this result may hold when considering multiplex ecological networks. Together, the small sets of interaction types in which species engage with each other and the astonishingly limited set of multiplex functional groups seems to reflect predictable evolutionary and ecological constraints operating in this entangled bank of species. This opens up a pathway toward simplifying ecosystem complexity into basic building blocks.

Previous theoretical studies have suggested that the incorporation of non-trophic interactions in food webs can have important consequences for species diversity [1,5,7], overall productivity [1], frequency of functional extinctions [39], stability [6,20,40–42], and the complexity–stability relationship [6,40,43]. May’s pioneering work in the early 1970s already included several interaction types [44]. Combining trophic and competitive interactions and using community matrices derived from real food webs, Yodzis [42] showed that a certain level of intraspecific interference contributed to the local stability of ecological communities, whereas interspecific competition tended to be destabilizing. In recent extensions of May’s work, Allesina and Tang [40] showed that matrices including mixtures of competition and mutualism were less likely to be locally stable than predator–prey matrices. Using a similar approach, Mougi and Kondoh [6] found that introducing a small proportion of mutualistic links could destabilize an otherwise stable food web, but that stability reached a peak at a moderate mixture of both interaction types (but see [45]). Studies on bipartite networks have suggested that the way different bipartite networks (e.g., mutualistic and antagonistic networks) are connected to each other could affect their stability [5]. Our study extends these results to show that the specific three-dimensional signature of the clusters and, in particular, the non-randomness of non-trophic interactions, can promote higher species persistence, higher total biomass, and higher robustness to extinctions than random networks in which the multidimensional connectivity pattern is lost.

A long history of theoretical and empirical work on food webs highlighted the importance not only of the structure of food webs (i.e., the repartition of the links in the web) [42,46–48] but also of the specific pattern of interaction strength for the stability of ecological communities [18,19,49]. Here, with the exception of a few common links, we lack information about interaction strengths for the entire Chilean web and especially about the strength of the non-trophic links. Getting information about those interaction strengths, their structure, the way they should be modeled, and their functional relevance remains an important empirical but also theoretical challenge.

To what extent the connectivity patterns identified in the Chilean web are unique to this intertidal community or general to all marine organisms or even to all ecosystems must be evaluated by comparison to those other ecosystems as more data on multiplex ecological networks becomes available [13,14,50]. The five functional groups identified could very well correspond to sets of strategies largely generalizable to other ecosystems. For example, a cluster of mobile consumers (top predators) might generally emerge. In the same vein, a group of sessile edible species competing for space is probably identifiable in many ecosystems. In terrestrial ecosystems, such a group would mostly be composed of basal primary producers, whereas in marine systems it could include sessile animals and exclude some primary producers that are not sessile (e.g., phytoplankton). Groups of sessile species that create biotic structure and habitat for others—notably, mobile consumers—while also competing for space are likely to be common across many ecosystems. Finally, identifying “multiplex hubs” in other ecosystems—such as mussels in the Chilean web, which create structure while also being an important food source—may help target a small subset of species that play a disproportionately important role for the community resilience. Conversely, some groups may be unique to marine benthic systems, such as the group of sessile, inedible scavengers formed here by anemones. It is noteworthy that a number of key groups of species are absent from the current version of the dataset (Materials and Methods). In particular, parasites are not included in the web. Studies have shown that food webs that also take parasites into account have increased connectivity and longer food chains, and the parasite–host links dominate numerically over predator–prey links [12]. Detritus (and thereby decomposers) are known to play an important role for the dynamics and structure of many communities and may also affect their stability [49,51,52]. It is unclear, however, what the significance is of local nutrient recycling in benthic marine and stream communities. In any case, adding missing species into the Chilean web could, depending on the connectivity of the newly introduced species, lead to either the emergence of new functional groups or the splitting of some of the current functional groups into additional groups [15].

The spatial and temporal variations of the patterns identified in the Chilean web remain to be investigated. This variability in space and time has been suggested to be essential to the stability and function of ecosystems [53]. The role of space may be particularly relevant in intertidal communities where mobile species (mainly consumers) could connect distant communities along the shore, with possible important consequences for the stability of these communities [48,53]. In addition, the incorporation of several interaction types in spatial ecology frameworks has been shown to have important consequences for community dynamics. For instance, Lurgi et al. [41] showed, using a spatially explicit individual-based model, that an increasing proportion of mutualistic links in a food web positively affected the dynamic stability of model communities.

How the topological structure of multiplex ecological networks modulates the multi-species dynamics and the resilience of ecosystems to perturbations, such as climate change, must be further investigated through other datasets [15], further dynamical modelling [1,5,20,41,45], and other approaches incorporating link weighting [3]. Until then, our results will help us guide future empirical studies and move toward a more general theory of how to leverage the full diversity of species interactions for understanding and predicting the dynamics and resilience of complex ecological systems.

## Materials and Methods

### The Dataset: The Chilean Web

The dataset [14] includes all the species that were found to co-occur during community structure surveys carried out at several rocky intertidal sites with similar wave exposure spread along 700 km of the central Chilean coast ([27], see [54,55] for sampling details and species list). Construction of the network was based on expert knowledge [14]. An interaction was included in the network if one species plausibly had a direct measurable effect on the growth, survival, or feeding rates of another species over an ecologically relevant time period (1–2 y) [14]. The dataset does not include parasites, endo-symbionts, or decomposers, because such data was unavailable for that community.

The network was split into three separate matrices for trophic, positive non-trophic, and negative non-trophic interactions (in each matrix, interactions are coded as 0 or 1) [14]. As a live and continuously improving network, some changes have been made to the network since first published [14]. These are mostly taxonomic changes and the inclusion of porcellanid crabs as part of the wave-exposed network. Furthermore, the biofilm taxa and plankton (zooplankton and phytoplankton) were each considered as a single node in the Chilean web due to lack of information.

The main assumptions made to build this network as well as possible related bias are discussed in Appendix A of [14]. In particular, we acknowledge that there may be “a bias in favor of negative non-trophic interactions at lower trophic levels,” because “measuring the relative importance of interference competition among rare species under natural conditions is particularly challenging” [14]. “When local experimental information was lacking for a pair of sessile species, we probably had a greater tendency in assigning (i.e., benefit of doubt) the interaction to competition for space than when dealing with pairs of mobile species at higher trophic levels. This would create a bias in favor of negative non-trophic interactions at lower trophic levels. However, the sheer number of species at bottom versus high trophic levels would make it difficult to alter the general pattern” [14].

Data deposited in the Dryad repository: http://dx.doi.org/10.5061/dryad.b4vg0 [21].

### Pairwise Multiplex Interactions

The pairwise multiplex interactions observed in the Chilean web were compared to those observed in random multiplex networks simulated layer by layer. For each layer, we imposed that the expected in- and out-degree sequences were equal to the degree sequences in the original layer of the Chilean web. To do so, we used the procedure explained in the “random network” paragraph hereafter. We calculated the statistical significance of any observed number of links by computing the empirical distribution of the number of links in the 10^4^ random multiplex networks.

### The Multiplex Probabilistic Clustering Algorithm

How can we tell what a multiplex network looks like? How can we summarize its structure? To answer these questions, classical approaches consist of pooling nodes that have similar connectivity patterns into clusters to extract the high-level structure of a complex network. Most of these approaches rely on finding modules or communities (clusters of nodes that are more connected inside than outside their cluster [56]). But, in ecological networks, could there be relevant structural patterns that we do not find because we have not thought to search beyond the modular structure? To circumvent this problem, we used a probabilistic clustering approach based on Stochastic block models [57–59]. Here, the cluster identification does not rely on any a priori hypothesis about the connectivity patterns to be found but aims precisely at identifying significant hidden connectivity patterns (e.g., modularity, centrality, hierarchy) or combinations of these patterns. Stochastic block models have been widely used for networks with one layer (see [30,60] for ecological networks), but not for multiplex networks as proposed in this paper. We followed the notations and the estimation procedure previously described in [60,61] and extended the model to multiplex networks with 3D-interactions using an appropriate probability distribution. The use of a probability distribution allows us to account for the randomness and the variability of the network and ensures a significant robustness to potential errors (spurious or missing links, for instance). We consider *n* = 106 interacting species, with Y_ij_ standing for the observed measure of these 3D interactions and Y = (Y_ij_). Y_ij_ is a 3-dimensional vector such that Y_ij_ = (Y_ij1_,Y_ij2_, Y_ij3_), where Y_ij1_ = 1 if there is a trophic interaction from *i* to *j* and 0 otherwise, Y_ij2_ for a positive interaction, and Y_ij3_ for a negative interaction. We now introduce the vectors (Z_1_,…,Z_n_), where for each species *i* Z_iq_ are the component of vector Z_i_ such that Z_iq_ = 1 if *i* belongs to cluster *q* and 0 otherwise. We assume that there are Q clusters with proportions a = (a_1_,…,a_Q_) and that the number of clusters Q is fixed (Q will be estimated afterward; see below). In a Stochastic block model, the distribution of Y is specified conditionally to the cluster membership:

$$Z_{i}\sim \text{Multinomial}\left(1,a\right),Z_{j}\sim \text{Multinomial}\left(1,a\right)$$ (1)

$$Y_{ij}|Z_{iq}Z_{jl}=1\sim f\left(.,\theta_{ql}\right),$$ (2)

where the distribution f(.,θ_ql_) is an appropriate distribution for the Y_ij_ of parameters θ_ql_. The novelty here is to use a 3D-Bernoulli distribution [62] that models the intermingling connectivity in the three layers—trophic, positive non-trophic, and negative non-trophic interactions. The objective is to estimate the model parameters and to recover the clusters using a variational expectation–maximization (EM) algorithm [60,63]. It is well known that an EM algorithm’s efficiency is governed by the quality of the initialization point. We propose to use the clustering partition obtained with the following heuristical procedure. We first perform a k-means clustering on the distance matrix obtained by calculating the Rogers and Tanimoto distance (R package ade4) between all the 3D interaction vectors V_i_ = (Y_i._,Y_.i_) associated to each species *i*. Second, we randomly perturb the k-means clusters by switching between 5 and 15 species membership. We repeat the procedure 1,000 times and select the estimation results for which the model likelihood is maximum. Lastly, the number of groups Q is chosen using a model selection strategy based on the integrated classification likelihood (ICL) (see S2 Fig) [61]. The algorithm eventually provides the optimal number of clusters, the cluster membership (i.e., which species belong to which cluster), and the estimated interaction parameters between the clusters (i.e., the probability of any 3D interaction between a species from a given cluster and another species from another or the same cluster). Source code (R/C++) is available upon request for people interested in using the method. See S1 Text for a discussion about the choice of this approach.

### The Dynamical Model

We use the bioenergetic consumer-resource model found in [32,64], parameterized in the same way as in previous studies [28,32,64–66], to simulate species dynamics. The changes in the biomass density *B*_*i*_ of species *i* over time is described by:

$$\frac{dB_{i}}{dt}=r_{i}\left(1-\frac{B_{i}}{K_{i}}\right)B_{i}+e_{i}B_{i}\sum_{j}F_{ij}TR\left(i,j\right)-\sum_{k}F_{ki}B_{k}TR\left(k,i\right)-x_{i}B_{i}$$ (3)

where *r*_*i*_ is the intrinsic growth rate (*r*_*i*_ >0 for primary producers only), *K*_*i*_ is the carrying capacity (the population size of species *i* that the system can support), *e* is the conversion efficiency (fraction of biomass of species *j* consumed that is actually metabolized), *F*_*ij*_ is a functional response (see Eq 4), TR is a n*n matrix with *n* the number of nodes in the network and whose *i*,*j* element is positive if species *i* consumes species *j*, and *x*_*i*_ is the metabolic rate.

The functional response of species *i* consuming species *j* is defined as multi-prey Holling-type functional response [67]:

$$F_{ij}=\frac{w_{i}b_{ij}B_{j}^{1+q}}{1+w_{i}h_{i}\sum_{k}TR\left(i,k\right)b_{ik}B_{k}^{1+q}}$$ (4)

where *w*_*i*_ is the relative consumption rate, which accounts for the fact that a consumer has to split its consumption between its different resources; it is defined as 1/(number of resources of species *i*), *b*_*ij*_ is the attack rate of predator *i* on prey *j*, *h*_*i*_ is the handling time of predator *i*, 1+*q* is the Hill-exponent with *q* the Hill coefficient (*q* = 0 yields a type II functional response, *q* = 1 yields a type III functional response).

#### Incorporation of the non-trophic interactions

The Chilean web encompasses a number of non-trophic interactions. The non-trophic links are stored in n*n matrices (with *n* the number of nodes in the network), whose *i*,*j* element is positive if species *i* has a non-trophic effect of that type on species *j*. Negative non-trophic links split into: competition for space (matrix COMP), predator interference (matrix INT), and increased mortality (or metabolism; matrix MORT). Positive non-trophic links can be split into improved recruitment (matrix REC), refuge provisioning (matrix REF) from predators, and increased survival (matrix FAC). As a first step in modeling these interactions, we introduced simple modifiers of the vital rates of target species (usually a saturation function).

Competition for space among sessile primary producers of the web is introduced by multiplying their growth term by a competition term as follows:

$$g_{i}=1-\sum_{k}COMP\left(k,i\right)c_{ki}B_{k}$$ (5)

where *k* refers to all the species competing for space with species *i* and *c*_*ki*_ is the intensity of competition between species *k* and *i*.

Predator interference is a negative non-trophic interaction that modifies the feeding of species *i* because of direct interactions with other predator species of the same prey. Previous studies have introduced it in the functional response as follows [68,69]:

$$F_{ij}=\frac{w_{i}b_{ij}B_{j}^{1+q}}{1+\sum_{l}INT\left(l,i\right)d_{li}B_{l}TR\left(l,j\right)+w_{i}h_{i}\sum_{k}TR\left(i,k\right)b_{ik}B_{k}^{1+q}}$$ (6)

where *l* is the other predators of prey *j*, and *d*_*li*_ is the interference term among the different predators of prey *j*.

Improved recruitment was incorporated into the growth term of primary producers (*r*_*i*_ in Eq 3) by saying that this term becomes a saturating function of the biomass of the facilitating species [1]:

$$r_{i_{new}}=\frac{r_{i}+r_{max_{i}}\sum_{k}REC\left(k,i\right)B_{k}}{1+\sum_{k}REC\left(k,i\right)B_{k}}$$ (7)

where *k* is the set of species improving the recruitment of species *i*, and *r*_*maxi*_ is the maximum growth (recruitment) rate reached in the presence of facilitators.

Refuge provisioning happens when a prey *j* is protected from its predator *i* by species *k*. It is incorporated in the attack rate *b*_*ij*_ as follows [1]:

$$b_{ij_{new}}=\frac{b_{ij}+b_{min_{ij}}\sum_{k}REF\left(k,j\right)B_{k}}{1+\sum_{k}REF\left(k,j\right)B_{k}}$$ (8)

where *k* the set of facilitators of species *j*, and *b*_*minij*_ is the minimum consumption reached in the presence of facilitators.

Positive and negative effects on survival were incorporated as follows [1]:

$$x_{i_{new}}=x_{i}-\frac{\left(x_{i}-x_{min_{i}}\right)\sum_{l}FAC\left(l,i\right)B_{l}}{1+\sum_{l}FAC\left(l,i\right)B_{l}}+\frac{\left(x_{max_{i}}-x_{i}\right)\sum_{k}MOR\left(k,i\right)B_{k}}{1+\sum_{k}MOR\left(k,i\right)B_{k}}$$ (9)

where *l* is the set of facilitators of *i* (whose presence contributes to increasing survival), *k* is the set of competitors of *i* (whose presence contributes to decreasing survival), *x*_*mini*_ is the minimum mortality reached in the presence of facilitators, and *x*_*maxi*_ is the maximum mortality reached in the presence of competitors.

The complete dynamical equation including non-trophic interactions can be written as:

$$\frac{dB_{i}}{dt}=r_{i_{new}}g_{i}\left(1-\frac{B_{i}}{K_{i}}\right)B_{i}+eB_{i}\sum_{j}F_{ij}TR\left(i,j\right)-\sum_{k}F_{ki}B_{k}TR\left(k,i\right)-x_{i_{new}}B_{i}$$ (10)

#### Simulations

Simulations were run in R using the ode function of the DeSolve library with the default integrator, lsoda. The model included 14 nodes (*n* = 14), which corresponded to the 14 clusters identified in the Chilean web (a species here is a “typical” species with 3D connectivity and biomass corresponding to the average inside the cluster). In this 14-species web, the links between two nodes (i.e., the values in the trophic and non-trophic matrices) are the frequency of interaction between clusters. Interactions among clusters are thus quantitative (between 0 and 1). Note that cluster 4 was replaced by plankton (i.e., a primary producer species) in the simulations. See S2 Table for the parameter values used. All simulations started with an initial biomass of 1 for all species. During simulations, species were considered to be extinct if their biomass *B*_*i*_ ≤ 10^−6^. Simulations were run for 2,000 time steps. We ran two sets of simulations. In the first set, the ecological web was initially fully intact. In the second set, one randomly selected species was removed from the ecological web. In both cases, we recorded total biomass and persistence, i.e., the number of species that remain at the end of a simulation. Simulations of the Chilean 14 species web were compared with simulations from 500 randomized networks (see next paragraph for how the random networks were generated).

### Random Networks

To test the significance of the assemblage of the different interaction types in the Chilean web, we simulated multiplex networks for which the most important topological properties (number of edges, in/out-degrees, degree correlation between layers) are identical to those in the Chilean web. For each layer (trophic, positive and negative non-trophic), we imposed that the expected in- and out-degree sequences (i.e., the list of species degrees) were equal to the degree sequences in the original layer of the Chilean web (S9 and S10 Figs and S1 Text). The consequence of these strong constraints is that (1) any species observed individually has the same 3-dimentional connectivity properties in the random networks, but is likely to have different partners than in the original Chilean web; and (2) the random networks are ecologically meaningful, because properties such as the trophic levels are conserved. Technically, we extrapolated the procedure in [70] and drew directed edges between species *i* and *j* with probability p_ij_ = (d_i_^out^ * d_j_^in^)/m, where m, d_i_^out^, and d_j_^in^ are the number of edges, the out-degree of *i*, and the in-degree of *j* in the given layer of the Chilean web. To avoid size effect biases, we only kept the simulated networks for which the number of edges is 100+/-2.5% the number of edges in the original Chilean web. For the pairwise analysis (Table 1), the three layers were randomized. For dynamical modeling, because we wanted to assess the role of the structure of the non-trophic interactions relative to the trophic one, the trophic layer was kept fixed and only the positive and negative non-trophic interaction layers were randomized.

#### Functional groups delimitation

The clusters gather species that are similar both in terms of their three-dimensional connectivity and in terms of the identity of the species they interact with. This raises the question of whether the network can be further aggregated into groups of clusters that have similar connectivity patterns beyond the identity of their interactors; in particular, different clusters can be similar because they gather species that are not involved in a specific kind of interaction (e.g., never the source of a positive link). We therefore calculated the Euclidian distance between the connectivity parameters (θ_q.,_θ_.q_) of all the pairs of the clusters identified. We then performed a hierarchical clustering (Ward’s method) on the obtained distance matrix: the principle consists in progressively merging the two (groups of) clusters that are the closest in terms of connectivity parameters. The cluster dendogram obtained shows the hierarchy of similarity between the clusters (i.e., the order of merging), which allows for identifying a higher degree of organization, hereafter referred to as “multiplex functional groups.”

#### Species attributes and functional groups

A regression tree analysis was used to explore the degree to which the multiplex functional groups could be explained by simple, easy-to-measure species traits that included shore height (ordinal), shore height breadth (ordinal), log (body mass), mobility (mobile versus sessile), and broad trophic level category (autotroph, herbivore, intermediate, top). A regression tree analysis is a non-parametric approach that recursively partitions the data into the most homogeneous subgroups. The threshold value at each split is determined computationally as the point of maximum discrimination between the two resulting subgroups.

#### Taxonomy and functional groups

We also explored whether taxonomic proximity between species explained functional group membership. We compiled the taxonomic information for 100 species from the WoRMS database (www.marinespecies.org), AlgaeBase (www.algaebase.org), and Macroalgal Herbarium Portal (http://macroalgae.org); we also manually added recovered taxonomic knowledge for six species. From this information, we built the cladogram and computed the patristic distance between all the species with the SeaView program (doua.prabi.fr/software/seaview). We calculated the statistical significance of the association between functional groups and taxonomy with a permutation test (10^5^ cluster membership permutations).

## Supporting Information

S1 Fig Observed number of pairwise multiplex links in the Chilean web for all possible types of multiplex links between a given pair of species. Nodes in black indicate species. Edges are blue, red, and gray for trophic, positive non-trophic, and negative non-trophic interactions, respectively. Two thousand, five hundred and ninety-six possible pairs of species in the web are not linked. Underlying data can be found in the Dryad repository: http://dx.doi.org/10.5061/dryad.b4vg0 [21].
(TIF)

S2 Fig Model log-likelihood (black) and integrated classification likelihood (ICL) criterion (red) for the Chilean web. Dashed line shows the ICL maximum for Q = 14 clusters. Underlying data can be found in the Dryad repository: http://dx.doi.org/10.5061/dryad.b4vg0 [21].
(TIF)

S3 Fig Cluster robustness to species extinction. Comparison between the multiplex clusters obtained with our probability algorithm for the Chilean web and for perturbed networks (obtained after driving part of the species of the original Chilean web to extinction). Agreement between clusters is assessed by: (left panel) the average adjusted Rand Index, aRI, whose value lies between 0 and 1, 1 being the value obtained for a perfect match between clusters (i.e., a perfect stability); and (right panel) the average number of clusters in the perturbed networks. The percentage of primary removed species (i.e., network nodes initially removed before the cascade of secondary extinctions) is indicated along the *x*-axis. Underlying data can be found in the Dryad repository: http://dx.doi.org/10.5061/dryad.b4vg0 [21].
(EPS)

S4 Fig Radial plots for the ingoing links of each cluster. Each radial plot shows the probability that there exists an incoming link between any node of a given cluster (upper numbers) to any node of the other clusters (numbers along the circle). Blue bars represent trophic links; black, negative non-trophic links; and red, positive non-trophic links. Underlying data can be found in the Dryad repository: http://dx.doi.org/10.5061/dryad.b4vg0 [21].
(TIF)

S5 Fig Radial plots for the outgoing links of each cluster (see legend of S4 Fig for more details). Underlying data can be found in the Dryad repository: http://dx.doi.org/10.5061/dryad.b4vg0 [21].
(TIF)

S6 Fig Alluvial diagrams comparing the clusters identified using the three-dimensional data to those of each of the layers independently (top row) or to those obtained using a combination of two of the three layers (bottom row). Top left: complete dataset versus trophic layer. Top middle: complete dataset versus negative non-trophic layer. Top right: complete dataset versus positive layer. Bottom left: complete dataset versus positive + negative non-trophic layers. Bottom middle: complete dataset versus trophic + negative non-trophic layer. Right: complete dataset versus trophic + positive non-trophic layer. Numbers in the boxes reflect arbitrary numbers given to the clusters (the numbers associated with the clusters of the complete dataset are the same as those used in the rest of the paper). Thickness of the box is related to the number of species in the cluster. Flows between the clusters show the species that are in common between the clusters (thickness of the flow is proportional to the number of species). Underlying data can be found in the Dryad repository: http://dx.doi.org/10.5061/dryad.b4vg0 [21].
(TIF)

S7 Fig Biomass variation after extinction of one species in the 14-species simulated networks (The *x*-axis corresponds to the ID of the cluster that the “species” in the network represents). The network whose topology is identical to the Chilean web is indicated by a red dot. Boxplots show the behavior of the 500 random networks. Biomass variation is calculated as (total biomass at steady state after extinction—total biomass at steady state before extinction)/(total biomass at steady state before extinction). Note that extinction of cluster 4 (plankton) is not simulated. Underlying data can be found in the Dryad repository: http://dx.doi.org/10.5061/dryad.b4vg0 [21].
(TIF)

S8 Fig Comparison of biomass and number of species observed after 2,000 time steps using either the structure of the Chilean web or one of the 500 random webs (see Materials and Methods) for a range of parameter values (12 values of INTNEG and INTPOS, 7 values for y and x0). Interpolation and heatmap were performed with the fields R package. Left: biomass *p*-value is the fraction of the 500 random networks for which the biomass is superior to the biomass of the Chilean web (in the dark blue areas, the biomass obtained using the structure of the Chilean web is significantly superior to the one obtained using random networks). Right: final number of species. The top row was plotted for x0 = 0.2227 and y = 10. The bottom row was plotted for INTPOS = 1 and INTNEG = 0.2. See S2 Table for other parameter values used for the simulations. Underlying data can be found in the Dryad repository: http://dx.doi.org/10.5061/dryad.b4vg0 [21].
(TIF)

S9 Fig Example of cumulative in/out degree distribution in the trophic, positive, and negative layers for the Chilean web (black) and for one random network obtained with our procedure explained in Materials and Methods. In this example, the three layers were randomized, as done for the pairwise analysis of Table 1. Underlying data can be found in the Dryad repository: http://dx.doi.org/10.5061/dryad.b4vg0 [21].
(EPS)

S10 Fig Cross-plot of the in/out degrees in the trophic, positive and negative layers, for the Chilean web (x axis) and for a random network (y axis) obtained with our procedure explained in Materials and Methods. Each point represents one species. Black line represents the perfect match between degrees in the Chilean web and in the random network. In this example, the 3 layers were randomized, as done for the pairwise analysis of Table 1. Underlying data can be found in the Dryad repository: http://dx.doi.org/10.5061/dryad.b4vg0 [21].
(EPS)

S11 Fig Cluster dendogram based on the distance between interaction parameters estimated by the probabilistic modeling for the different clusters identified. Rectangles illustrate the multiplex functional groups. Underlying data can be found in the Dryad repository: http://dx.doi.org/10.5061/dryad.b4vg0 [21].
(TIF)

S12 Fig Regression tree aiming at predicting the multiplex functional groups as a function of species attributes (R^2^ = 0.81). All species of the data set were considered except for the group “plankton” (i.e., 105 species). We used the following attributes to predict the multiplex functional groups: short height (ordinal score: low = 1, mid = 2, high = 3, low-mid = 1.5, etc.), shore height breadth (ordinal; “low-mid” = 2, “low” = 1, “low-mid-high” = 3), log body mass, mobility (mobile/sessile), trophic level category (basal, herbivore, intermediate, top). For each “leaf” in the tree, the horizontal bar shows the proportion of species in each functional group, while the number indicated below “count” is the number of species. The variable selected for each split is directly under the parent. Note: “basal” here refers to autotroph species. Underlying data can be found in the Dryad repository: http://dx.doi.org/10.5061/dryad.b4vg0 [21].
(TIF)

S1 Table Table displaying the layers used along the columns and the clusters along the rows. A cross indicates the layer (or combination of two layers) where the clusters are preserved (i.e., identical), compared to the case in which the whole dataset is used (i.e., the three layers of interactions; last column of the table). The minimum information required to obtain the cluster is in yellow. Underlying data can be found in the Dryad repository: http://dx.doi.org/10.5061/dryad.b4vg0 [21].
(DOCX)

S2 Table Parameter names, definitions, and values used for the simulations of the dynamical model (Figs 3, S7 and S8). Underlying data can be found in the Dryad repository: http://dx.doi.org/10.5061/dryad.b4vg0 [21].
(DOCX)

S1 Text (DOCX)

## Abbreviations

EM: expectation–maximization
ICL: integrated classification likelihood
