# Supplementary figures and images for: How Structured Is the Entangled Bank? The Surprisingly Simple Organization of Multiplex Ecological Networks Leads to Increased Persistence and Resilience
Source: PLoS Biol. 2016 Aug 3;14(8):e1002527. doi: 10.1371/journal.pbio.1002527 (PMC4972357; doi:10.1371/journal.pbio.1002527)

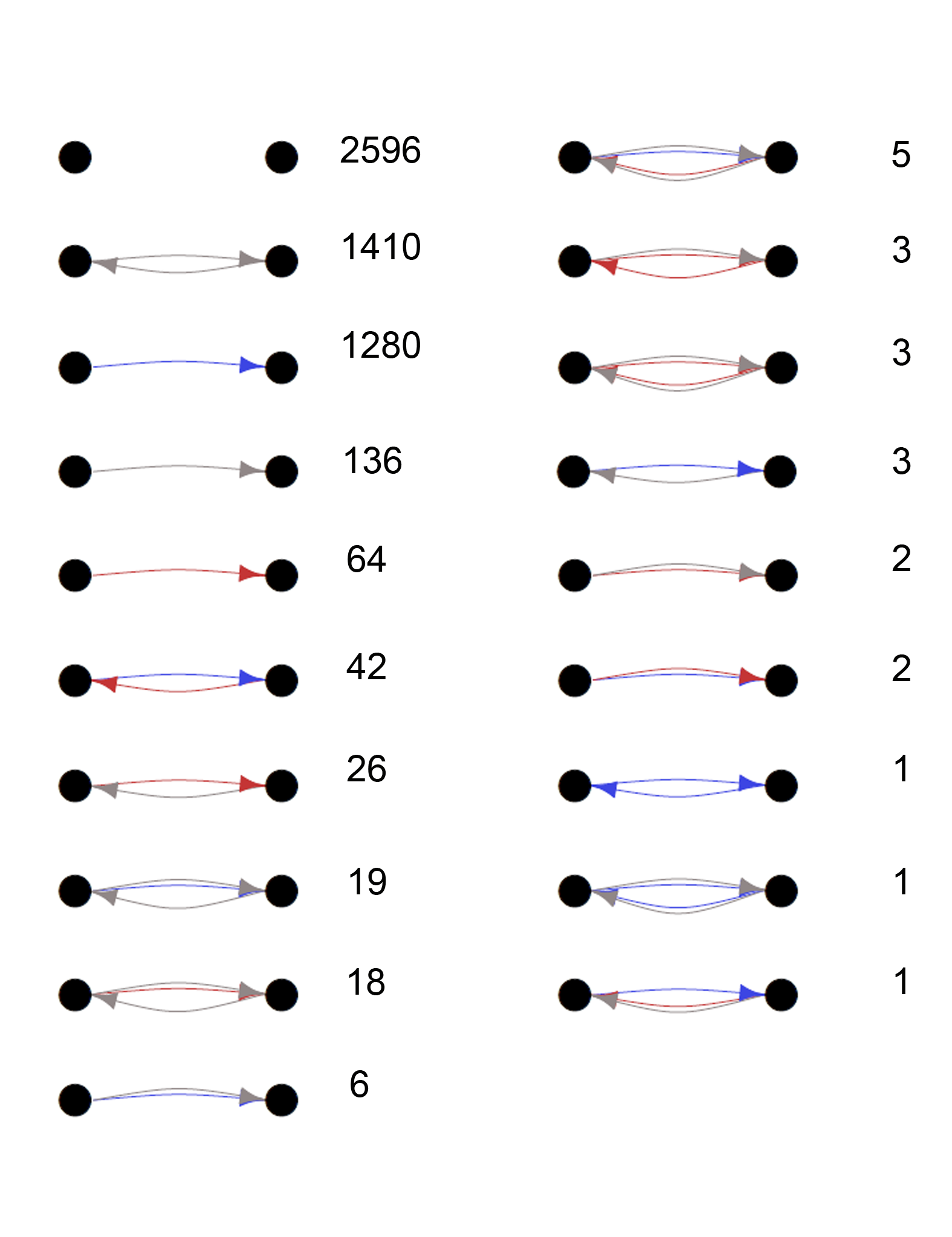

Supplement: S1 Fig — Nodes in black indicate species. Edges are blue, red, and gray for trophic, positive non-trophic, and negative non-trophic interactions, respectively. Two thousand, five hundred and ninety-six possible pairs of species in the web are not linked. Underlying data can be found in the Dryad repository: http://dx.doi.org/10.5061/dryad.b4vg0 [21]. (TIF) [file pbio.1002527.s001.tif]

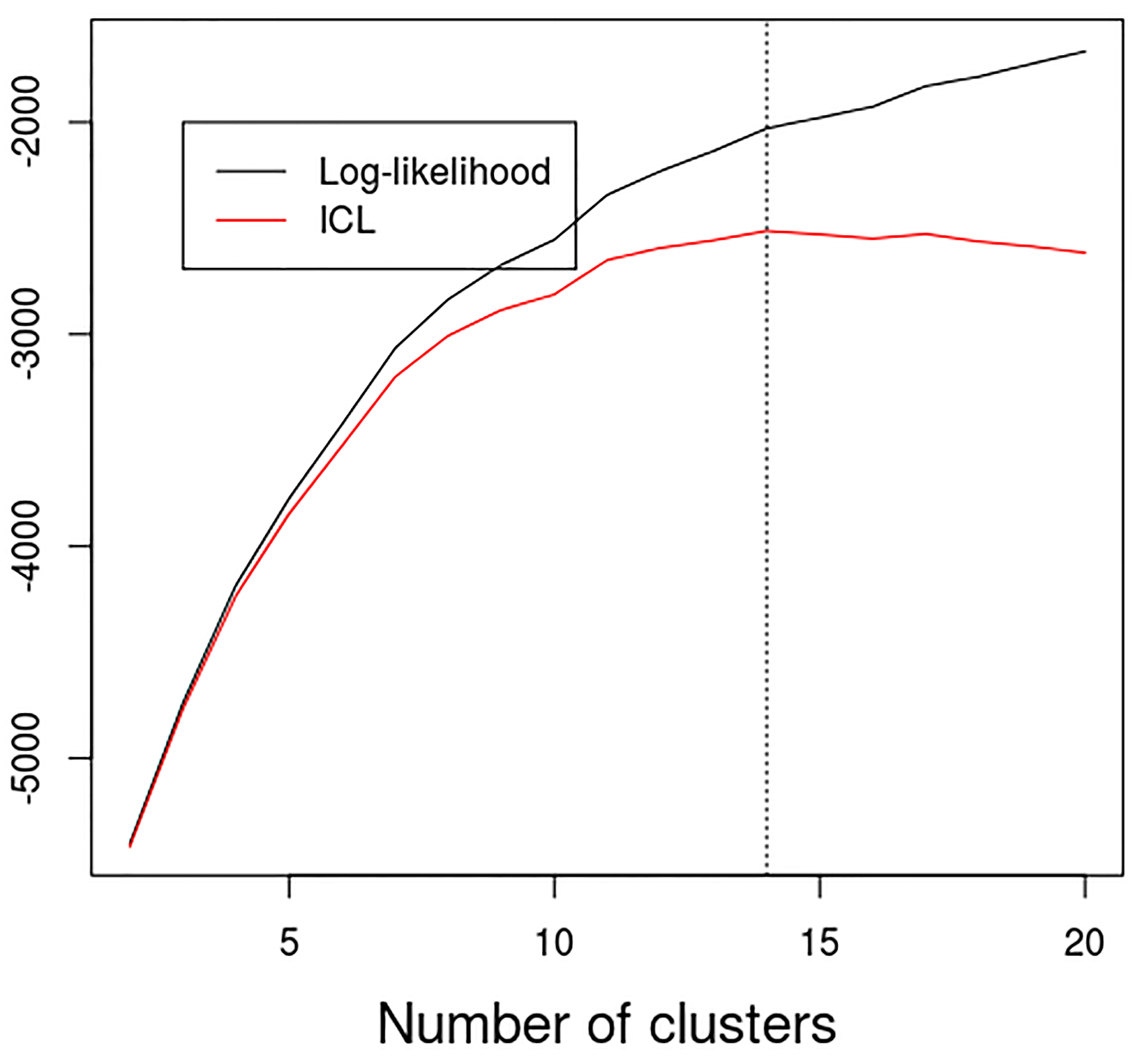

Supplement: S2 Fig — Dashed line shows the ICL maximum for Q = 14 clusters. Underlying data can be found in the Dryad repository: http://dx.doi.org/10.5061/dryad.b4vg0 [21]. (TIF) [file pbio.1002527.s002.tif]

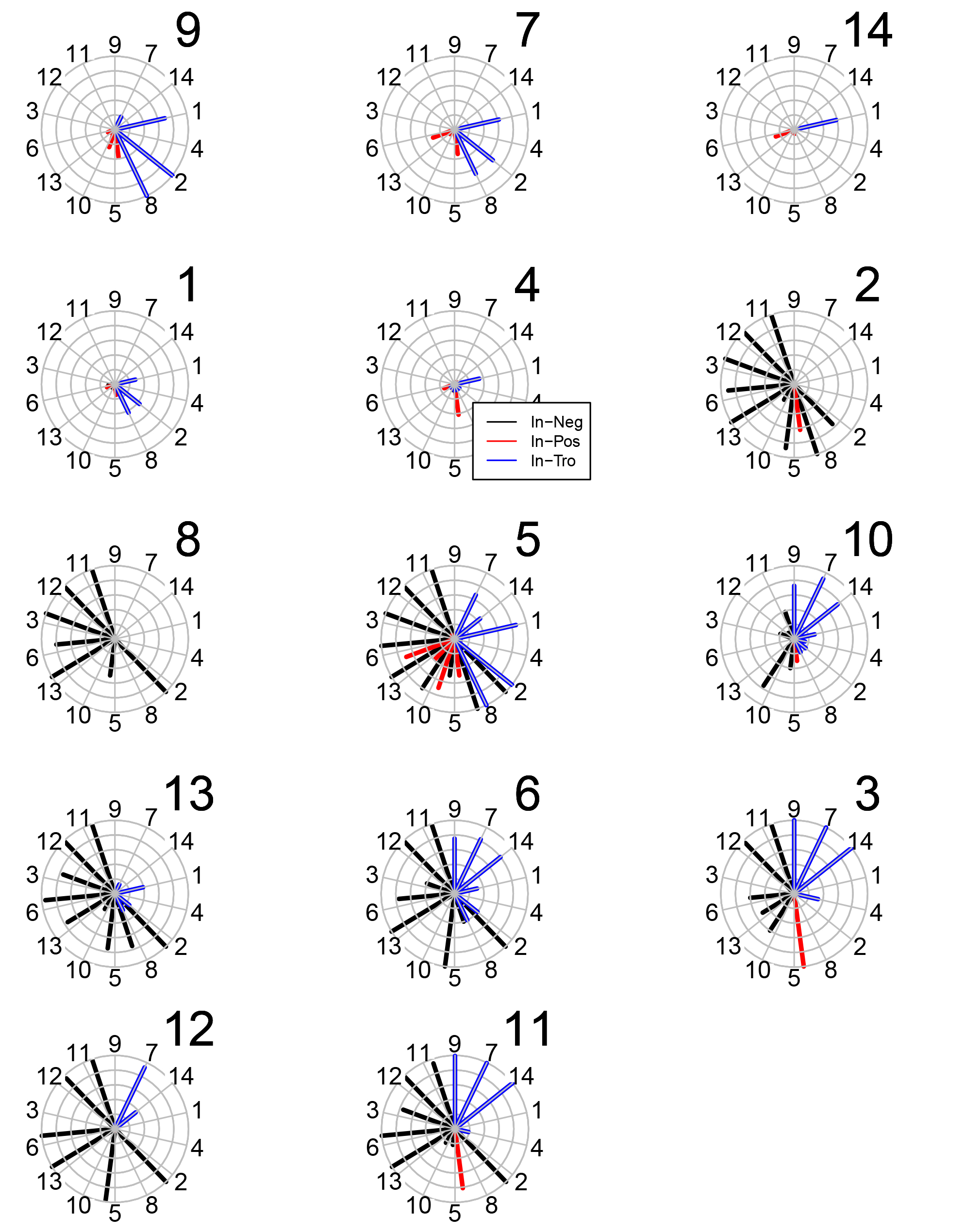

Supplement: S4 Fig — Each radial plot shows the probability that there exists an incoming link between any node of a given cluster (upper numbers) to any node of the other clusters (numbers along the circle). Blue bars represent trophic links; black, negative non-trophic links; and red, positive non-trophic links. Underlying data can be found in the Dryad repository: http://dx.doi.org/10.5061/dryad.b4vg0 [21]. (TIF) [file pbio.1002527.s004.tif]

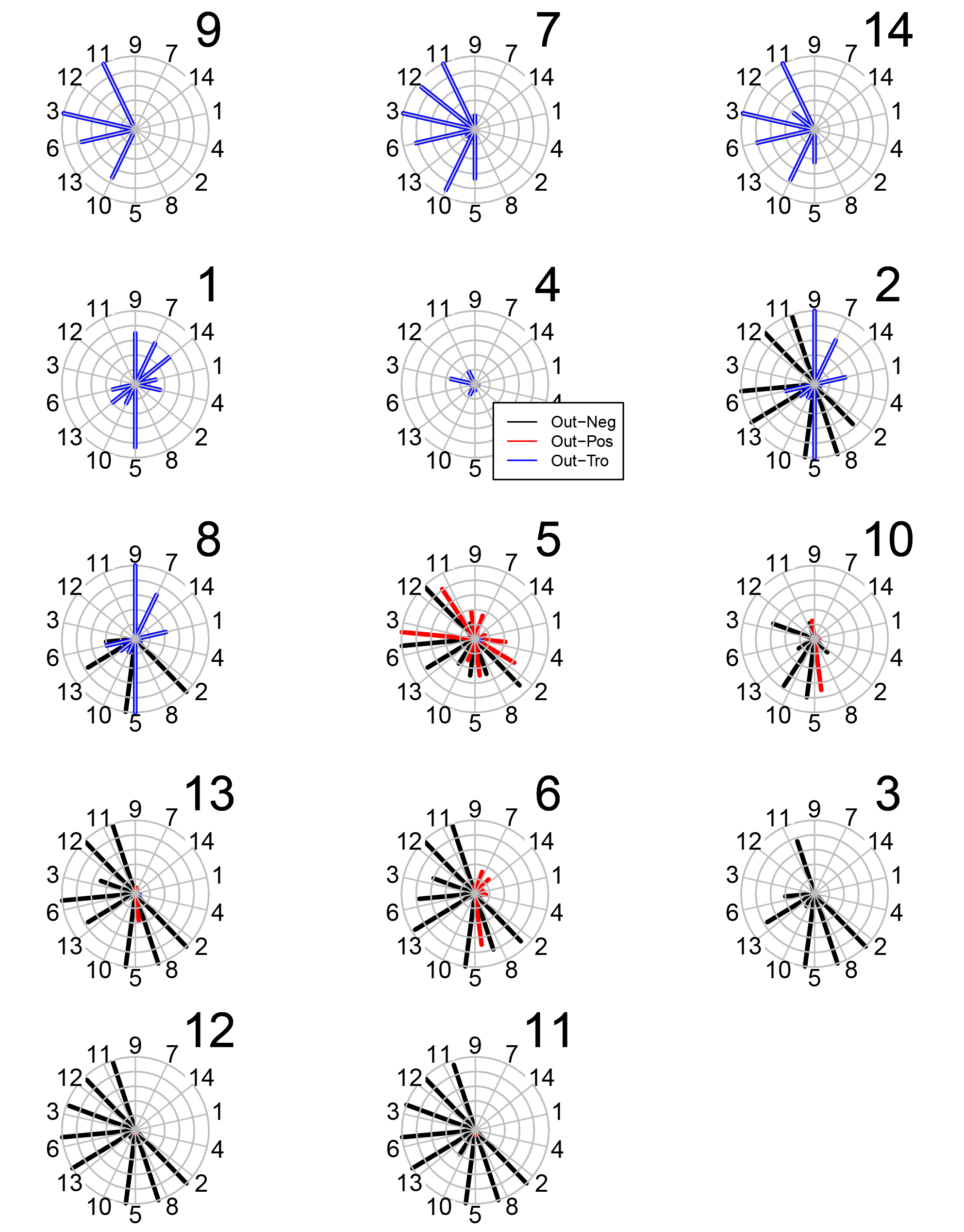

Supplement: S5 Fig — Underlying data can be found in the Dryad repository: http://dx.doi.org/10.5061/dryad.b4vg0 [21]. (TIF) [file pbio.1002527.s005.tif]

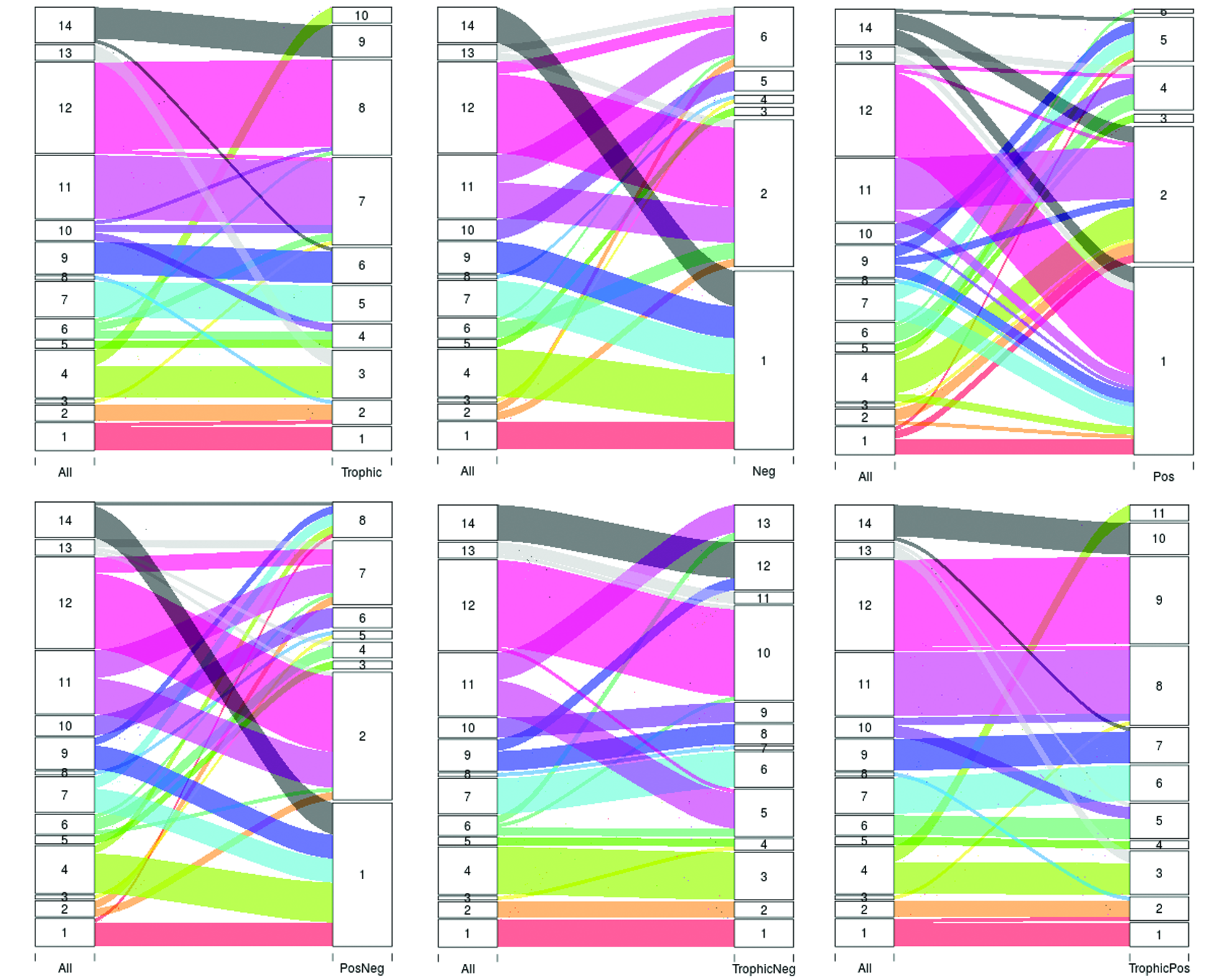

Supplement: S6 Fig — Top left: complete dataset versus trophic layer. Top middle: complete dataset versus negative non-trophic layer. Top right: complete dataset versus positive layer. Bottom left: complete dataset versus positive + negative non-trophic layers. Bottom middle: complete dataset versus trophic + negative non-trophic layer. Right: complete dataset versus trophic + positive non-trophic layer. Numbers in the boxes reflect arbitrary numbers given to the clusters (the numbers associated with the clusters of the complete dataset are the same as those used in the rest of the paper). Thickness of the box is related to the number of species in the cluster. Flows between the clusters show the species that are in common between the clusters (thickness of the flow is proportional to the number of species). Underlying data can be found in the Dryad repository: http://dx.doi.org/10.5061/dryad.b4vg0 [21]. (TIF) [file pbio.1002527.s006.tif]

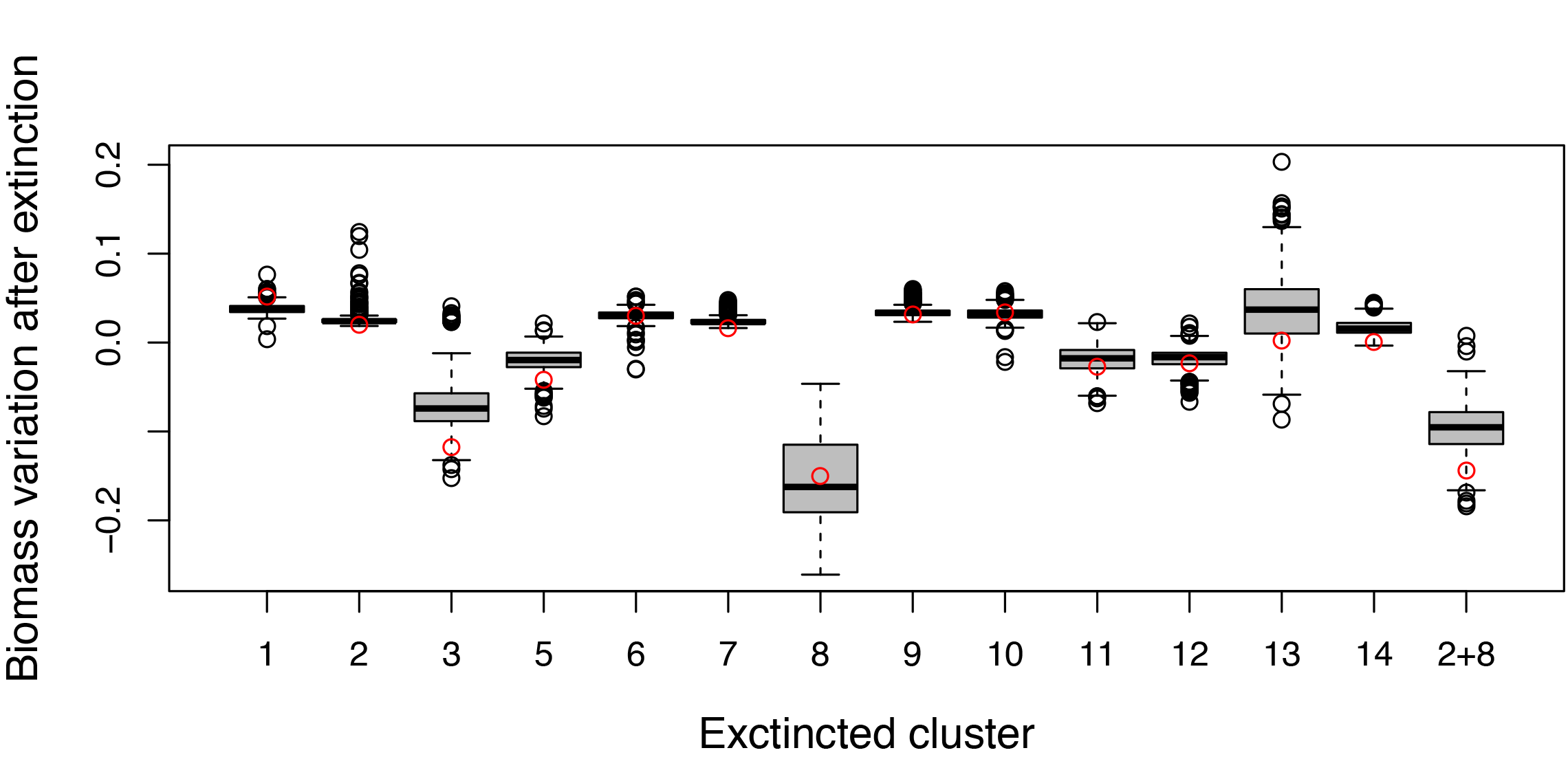

Supplement: S7 Fig — The network whose topology is identical to the Chilean web is indicated by a red dot. Boxplots show the behavior of the 500 random networks. Biomass variation is calculated as (total biomass at steady state after extinction—total biomass at steady state before extinction)/(total biomass at steady state before extinction). Note that extinction of cluster 4 (plankton) is not simulated. Underlying data can be found in the Dryad repository: http://dx.doi.org/10.5061/dryad.b4vg0 [21]. (TIF) [file pbio.1002527.s007.tif]

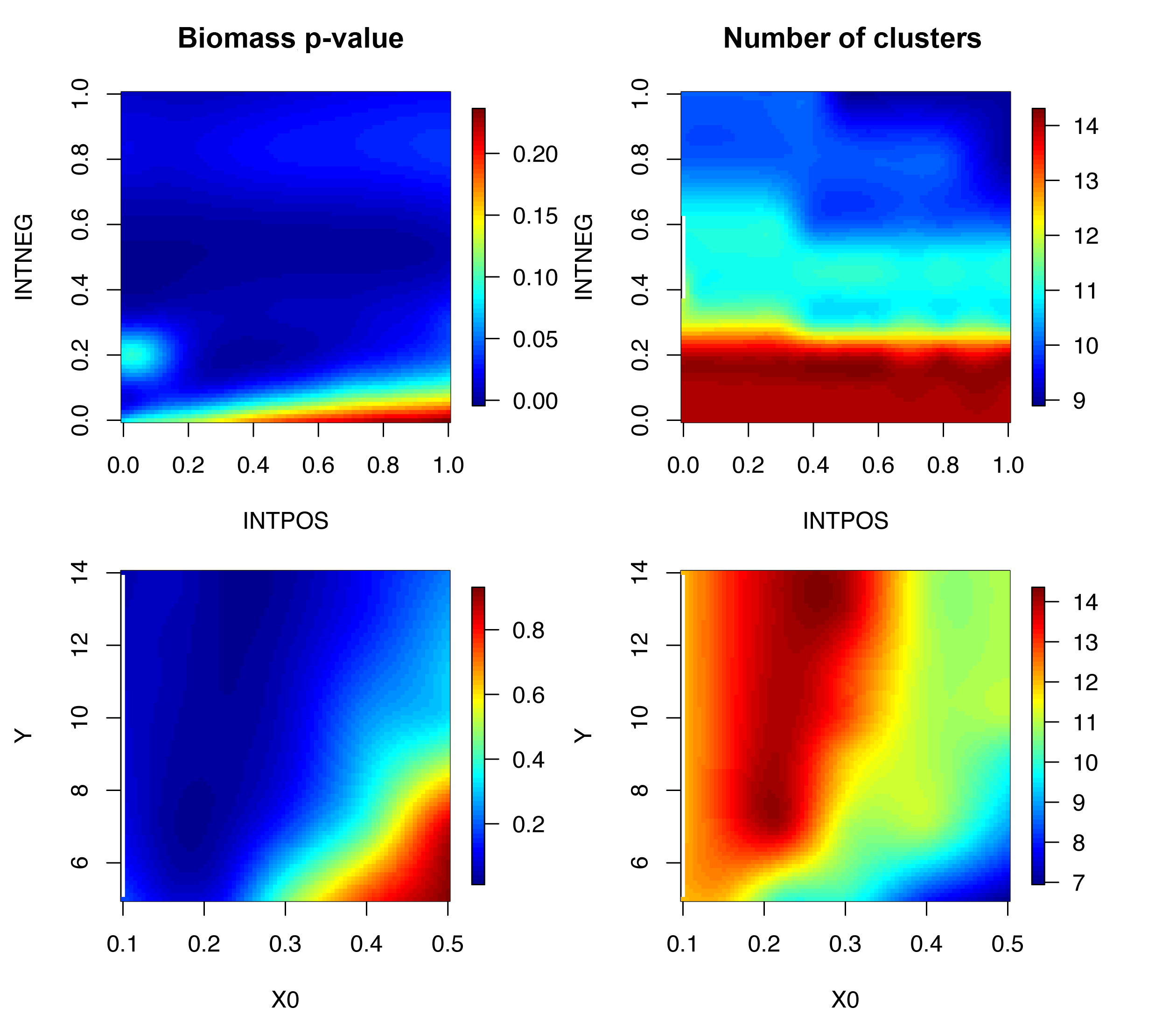

Supplement: S8 Fig — Interpolation and heatmap were performed with the fields R package. Left: biomass p-value is the fraction of the 500 random networks for which the biomass is superior to the biomass of the Chilean web (in the dark blue areas, the biomass obtained using the structure of the Chilean web is significantly superior to the one obtained using random networks). Right: final number of species. The top row was plotted for x0 = 0.2227 and y = 10. The bottom row was plotted for INTPOS = 1 and INTNEG = 0.2. See S2 Table for other parameter values used for the simulations. Underlying data can be found in the Dryad repository: http://dx.doi.org/10.5061/dryad.b4vg0 [21]. (TIF) [file pbio.1002527.s008.tif]

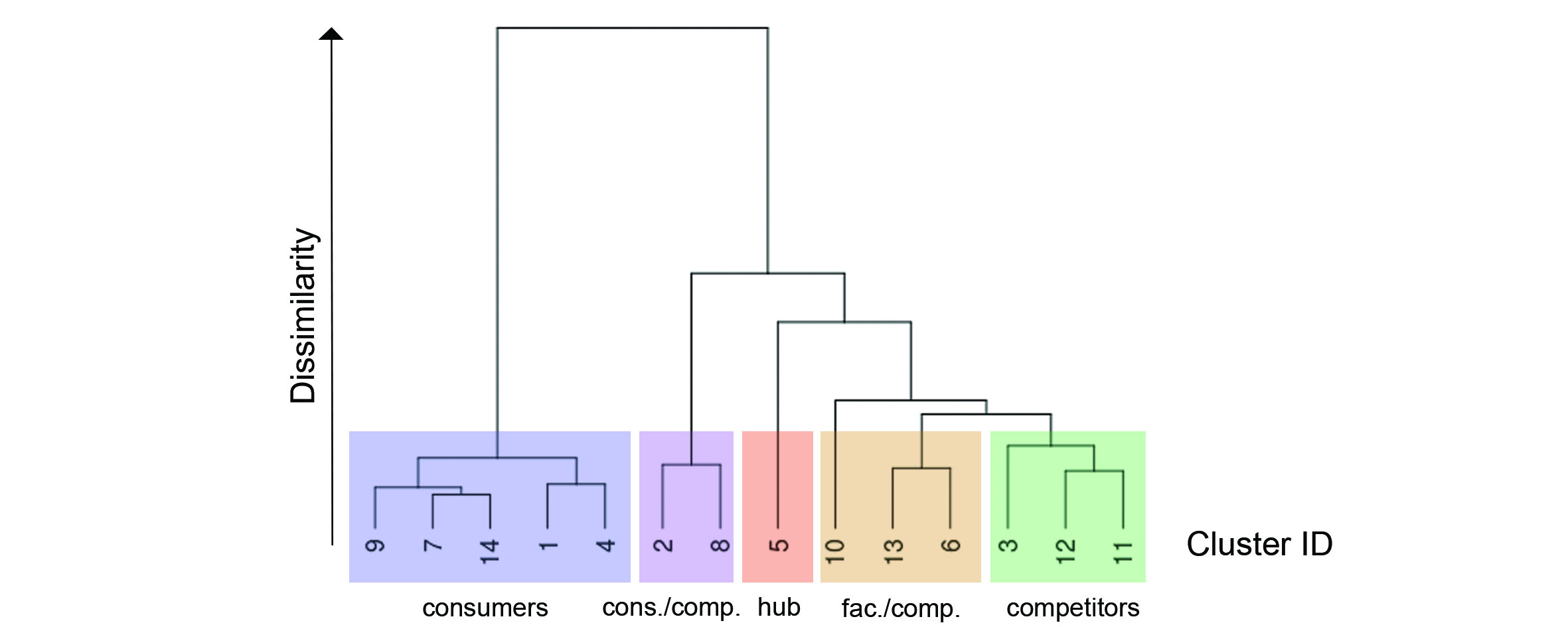

Supplement: S11 Fig — Rectangles illustrate the multiplex functional groups. Underlying data can be found in the Dryad repository: http://dx.doi.org/10.5061/dryad.b4vg0 [21]. (TIF) [file pbio.1002527.s011.tif]

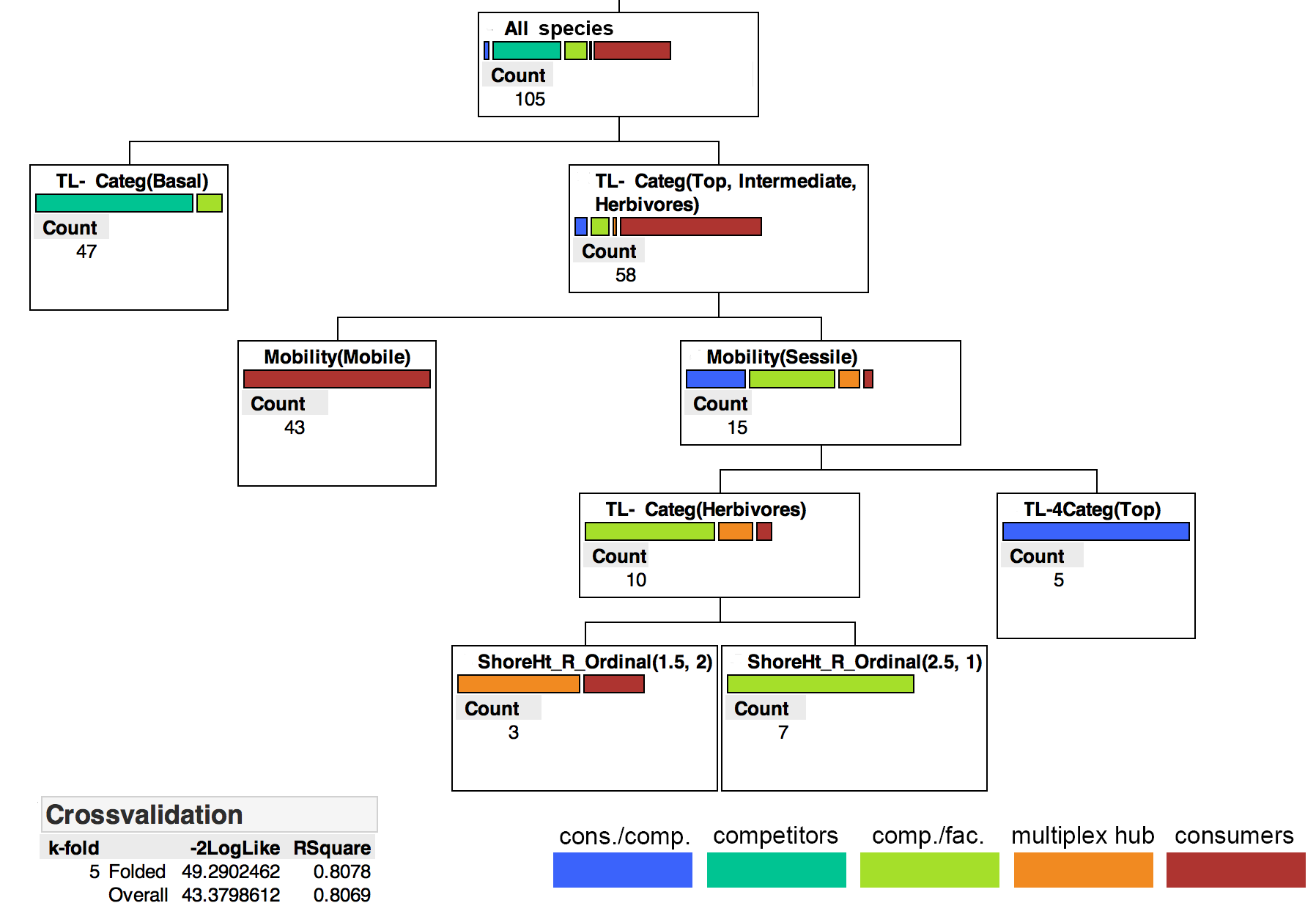

Supplement: S12 Fig — All species of the data set were considered except for the group “plankton” (i.e., 105 species). We used the following attributes to predict the multiplex functional groups: short height (ordinal score: low = 1, mid = 2, high = 3, low-mid = 1.5, etc.), shore height breadth (ordinal; “low-mid” = 2, “low” = 1, “low-mid-high” = 3), log body mass, mobility (mobile/sessile), trophic level category (basal, herbivore, intermediate, top). For each “leaf” in the tree, the horizontal bar shows the proportion of species in each functional group, while the number indicated below “count” is the number of species. The variable selected for each split is directly under the parent. Note: “basal” here refers to autotroph species. Underlying data can be found in the Dryad repository: http://dx.doi.org/10.5061/dryad.b4vg0 [21]. (TIF) [file pbio.1002527.s012.tif]
