# Supplementary material for: How Structured Is the Entangled Bank? The Surprisingly Simple Organization of Multiplex Ecological Networks Leads to Increased Persistence and Resilience
Source: PLoS Biol. 2016 Aug 3;14(8):e1002527. doi: 10.1371/journal.pbio.1002527 (PMC4972357; doi:10.1371/journal.pbio.1002527)
Supplement: S1 Table — A cross indicates the layer (or combination of two layers) where the clusters are preserved (i.e., identical), compared to the case in which the whole dataset is used (i.e., the three layers of interactions; last column of the table). The minimum information required to obtain the cluster is in yellow. Underlying data can be found in the Dryad repository: http://dx.doi.org/10.5061/dryad.b4vg0 [21]. (DOCX) [file pbio.1002527.s013.docx]

|  | T only | N only | P only | T+N | T+P | N+P | All |
| --- | --- | --- | --- | --- | --- | --- | --- |
| 14 | X |  |  |  |  |  | X |
| 9 | X |  |  |  |  |  | X |
| 7 | X |  |  | X | X |  | X |
| 4 |  |  |  | X |  |  | X |
| 1 |  |  |  | X |  |  | X |
| 6 |  |  |  |  |  |  | X |
| 10 |  | X |  | X |  | X | X |
| 13 |  |  |  |  |  |  | X |
| 3 |  |  |  |  |  |  | X |
| 11 |  |  |  |  |  |  | X |
| 12 |  |  |  |  |  |  | X |
| 2 |  |  |  | X |  |  | X |
| 8 |  |  |  | X |  |  | X |
| 5 |  | X | X |  |  | X | X |
