# Supplementary material for: How Structured Is the Entangled Bank? The Surprisingly Simple Organization of Multiplex Ecological Networks Leads to Increased Persistence and Resilience
Source: PLoS Biol. 2016 Aug 3;14(8):e1002527. doi: 10.1371/journal.pbio.1002527 (PMC4972357; doi:10.1371/journal.pbio.1002527)
Supplement: S2 Table — Underlying data can be found in the Dryad repository: http://dx.doi.org/10.5061/dryad.b4vg0 [21]. (DOCX) [file pbio.1002527.s014.docx]

| **Parameter** | **Definition** | **Value** |
| --- | --- | --- |
| **m_i_** | Body mass of species *i* | 1 for algae, median biomass of species in the cluster otherwise (see values in the text above the table) |
| **r_i_** | intrinsic growth rate of primary producer *i*  (*r_i_* >0 for primary producers only) | m_i_^(-0.25) for plants, 0 otherwise |
| **K_i_** | carrying capacity of species *i* | 1 [6,9] |
| **e** | conversion efficiency | 0.85 [9] |
| **x_i_** | metabolic rate of species *i* | 0.138 for primary producers [6]  otherwise : x0*m_i_^(-0.25)  with x_0_ = 0.2227 [5] for Fig. 3 and x_0_ varies between 0.1 and 0.5 for Fig. S8 [2,5,6]. |
| **w_i_** | relative consumption rate of species *i* | 1/(number of resources of species *i*) |
| ***b_ij_*** | attack rate of predator *i* on prey *j* | x_i_ *y/B_o_  with x_i_ the metabolic rate,  y the maximum consumption rate (y=10 for Fig. 3 [10] and y varies between 5 and 14 for Fig. S8 [2,3,5,6]),  B_o_=0.5 [6,7,9,10] the half-saturation density |
| **h_i_** | handling time of predator i | 1/(y* x_i_) with y =10 [10]  and x_i_ the metabolic rate |
| **1+q** | Hill exponent | 2  (type III functional response) |
| **q** | Hill coefficient | 1 |
| **INTPOS** | intensity of positive non-trophic interactions | 1 in Fig. 3, varies between 0 and 1 in Fig. S8 |
| **INTNEG** | intensity of negative non-trophic interactions | 0.2 in Fig. 3, varies between 0 and 1 in Fig. S8 |
| **c_ij_** | intensity of competition from *i* to *j* | INTNEG |
| **d_ij_** | interference from predator *i* on predator *j* | INTNEG |
| **r_maxi_** | maximum growth rate of species *i* reached in the presence of facilitators | (1+INTPOS)*r_i_ |
| **b_minij_** | Minimum attack rate of predator *i* on prey *j* reached in the presence of facilitators of the prey *j* | b_ij_/(1+INTPOS) |
| ***x_mini_*** | minimum mortality reached in the presence of facilitators | X_i_/(1+INTPOS) |
| ***x_maxi_*** | maximum mortality reached in the presence of competitors. | (1+INTNEG)*x_i_ |
