# Supplementary material for: How Structured Is the Entangled Bank? The Surprisingly Simple Organization of Multiplex Ecological Networks Leads to Increased Persistence and Resilience
Source: PLoS Biol. 2016 Aug 3;14(8):e1002527. doi: 10.1371/journal.pbio.1002527 (PMC4972357; doi:10.1371/journal.pbio.1002527)
Supplement: S1 Text — (DOCX) [file pbio.1002527.s015.docx]

Supporting Information

How structured is the entangled bank?

The surprisingly simple organization of multiplex ecological networks leads to increased persistence and resilience

Sonia Kéfi^☯*^, Vincent Miele^☯^, Evie A. Wieters, Sergio A. Navarrete_,_ Eric L. Berlow

^☯^These authors contributed equally to the study

1. **The multiplex probabilistic clustering algorithm**

In most existing methods based on block modelling [1–3], deterministic approaches have been used (i.e. partitioning algorithms to find blocks that minimize some criteria); in other terms, these studies did not assume a probabilistic model. The novelty of our approach here is not really in the model itself but in the idea of applying stochastic block models 1/ on an ecological data set (although a first stone was proposed in [4]) and 2/ for multiplex networks. We chose a model-based approach because 1/ the use of probability distributions (3D Bernouilli in our case) allows to account for the randomness and the variability of the network, in the sense that this is less stiff than exact partitioning approaches and it is more robust to potential errors (spurious or missing links), 2/ we can apply model selection theory in this framework for the choice of the number of clusters (still an open question for k-means method for instance), 3/ we can plug probability distributions that rely on covariates such as phenotypic (body size,...), space or phylogenetic information.

**Robustness of the clusters to species extinctions**

We evaluated the robustness of our clustering algorithm by checking how similar the identified clusters were after species extinctions in the network. We therefore simulated the extinctions of 5, 10, 15, 20, 25, 30, 40 and 50% of the species in the network. The species driven to extinction were randomly taken in the network (repeated 50 times for each of the percentages). A species removal was accompanied by the removal of its links and potentially by a cascade of secondary extinctions, i.e. the removal of other species that have lost all of their trophic outgoing links (species that have no prey or resource anymore). The resulting *perturbed networks* were analyzed with the same clustering algorithm, and the number of clusters and their species composition were compared to those retrieved from the original Chilean web. The agreement between the clusters obtained for the Chilean web and for a perturbed network was assessed by the adjusted Rand Index [5] that measures the agreement between two partitions. It lies between 0 and 1, 1 being the value obtained for a perfect match between clusters. The cluster number and their species composition was largely conserved after simulated extinctions of up to 30% of the species in the Chilean web (S3 Fig).

Since the clustering analysis gathers species that are similar in terms of their connectivity patterns, we expect species within a cluster to be largely redundant. Important for the secondary extinctions analysis of S3 Fig, this includes redundancy of trophic interactions. Therefore, the robustness of the cluster number and composition is not surprising, but it is a confirmation of the redundancy of species in the clusters and S3 Fig illustrates the extant of that redundancy.

1. **Comparison of the clusters obtained based on the different layers of interactions**

How does the grouping into clusters vary depending on the layer of the network used? It is expected to get a higher level of refinement of the clusters when additional layers are incorporated in the data, but does one of the layers contains already most (or all) of the information needed to define a given cluster? We applied the same probabilistic algorithm to each of the layers individually (or to the combination of two of the three layers), and we compared the clusters identified to those obtained when using the 3-dimentional information. The results are displayed in S6 Fig.

We obtained 14 clusters using all interaction types, 10 clusters with the trophic interactions only and 6 clusters when using the negative or the positive non-trophic interactions (S5 Fig, top row). We summarize in S1 Table in which of the layer (or combination of two layers) a given cluster is conserved. This reveals that 3 clusters from the consumers’ group (clusters 7, 9, 14) are conserved when only the trophic layer is used, two clusters (10 and 5) are conserved using the negative non-trophic layer only, two clusters (7 and 5) are conserved when using the positive layer only. The rest of the clusters (4, 1, 6, 13, 3, 11, 12, 2 and 8) require at least two layers (T+N for 4,1,2,8 and all layers for 6, 13, 3, 11 and 12).


In sum, none of the layers contains by itself enough information to retrieve the multiplex clusters. The trophic layer seems to contain already quite some information related to the cluster structure, but the addition of the non-trophic interactions considerably refines the definition of the clusters. In particular, we would not be able to identify and characterize the functional groups if it was not for the non-trophic interactions. Besides the clusters of the consumers’ functional groups, none of the other functional groups could be identified with the trophic layer only. Their distinction from the rest of the network relies on the non-trophic interactions (and more precisely their involvement in the combined 3 types of interactions). In particular, the cluster of mussels (cluster 5) only comes out when one of the non-trophic interaction layers is incorporated.

S6 Fig also suggests that if other types of interactions were added to the data set (e.g. parasites), the clusters identified would most likely be different. We would indeed probably get a more precise description of species multidimensional niche with more interactions. However, although we expect the clusters to be more detailed, we do not expect the functional groups to be altered, but this remains to be validated by future analysis as the data set will improve and other data sets will become available.

1. **The dynamical model**

See S2 Table for the parameter values used in the model simulations (model equations are described in Material and Methods). Parameter values were taken from [6–9]. Cluster 4 was replaced by plankton. The body mass used for the 14 clusters were 1 for the clusters of algae and the median body mass of the species of the clusters otherwise:

8.7, 10.9, 1, 0.01, 1.28, 1, 6, 11, 0.17, 1, 1, 1, 1.93, 6.14.

The matrix of interactions among clusters was derived from the probabilistic clustering approach. Thereby, the values in the matrices TR, REC, REF, FAC, MOR, COMP, INT are between 0 and 1.


**Sensitivity analysis**

For the whole range of possible intensity of positive and negative interactions, we ran simulations of the model for which we quantified to what extent the final total biomass and number of species obtained using the structure of the Chilean web differed from those obtained using the structure of 500 random networks (see Material and Methods; S8 Fig).

Results show that the biomass obtained using the structure of the Chilean web is significantly superior to the one obtained using random networks for a broad range of non-trophic intensities. This result is in particular true as soon as the intensity of non-trophic interactions (INTNEG) is above a threshold value which depends on the intensity of positive non-trophic interactions (INTPOS; see S8 Fig top left). The maximum number of species is obtained for intermediate values of negative non-trophic interactions (INTNEG around 0.2), and this independent of the intensity of the positive non-trophic interactions.

In the same vein, the biomass observed using the structure of the Chilean web is significantly superior than expected using random network structures for a broad range of metabolism and maximum consumption rates (resp. x_0_ and y; S8 Fig bottom left), and the maximum number of species is obtained for low to medium values of metabolism in a broad range of maximum consumption rate (S8 Fig bottom).

For Fig 3 in the main text, we chose to illustrate those results using a combination of parameter values for which 14 species could be maintained (x_0_=0.2227 and y=10; S8 Fig bottom right) and we chose the intensity of the non-trophic intensities at the edge of the significance area for the biomass (INTNEG=0.2, INTPOS=1; S8 Fig top left).

1. **Topology of the random networks**

The random networks keeping the same sequence of in and out degrees (see Materials and Methods) have very similar topological properties as those of the Chilean web (S9 and S10 Fig). In particular, the in/out degrees are (almost) equal in the real and simulated networks (see S9 and S10 Figs) for each of the three layers, meaning that it conserves the correlation between the degrees. This correlation between degrees is done at the node level but not at the level of pairs of species. Thus, the null model used is very strongly constrained.

This null model was used to calculate the expected number of pairwise interactions in Table 1 (one type, two types, all types) and for the dynamical model simulations (Fig 3, S7 and S8 Figs).

1. **Functional groups**

In S11 Fig, we gathered cluster 10 with clusters 6 and 13 because we empirically observed that cluster 10 also gathers facilitators/competitors species, even if the species of cluster 10 do not compete as much as those of cluster 6 and 13.

Note that the identification of the clusters and thereby of the functional groups is only based on the interactions, not the traits, and that the definition of the presence/absence of an interaction between a pair of species was based on knowledge about the interaction itself - and not on species traits (see Materials and Methods of the paper and [11] for more information about how the data set was assembled). Because species traits are determinant for interactions, it makes sense that the clusters found, which gather species that are similar in terms of their interaction patterns, also share similar traits and belong to close taxonomic groups. It makes sense, but it is not necessarily expected (see clusters 6 and 10). Moreover, there is no reason to think that only a few sets of traits could very well predict not only each of the three layers of interactions independently but in this case the three dimensions simultaneously.

**References**

1. Doreian P, Batagelj V, Ferligoj A. Generalized Blockmodeling. Cambridge University Press; 2004.

2. White HC, Boorman SA, Breiger RL. Social Structure from Multiple Networks. I. Blockmodels of Roles and Positions. Am J Sociol. 1976;81: 730–780.

3. Harrer A, Schmidt A. An Approach for the Blockmodeling in Multi-Relational Networks. 2012 IEEE/ACM International Conference on Advances in Social Networks Analysis and Mining (ASONAM). 2012. pp. 591–598. doi:10.1109/ASONAM.2012.100

4. Mariadassou M, Robin S, Vacher C. Uncovering latent structure in valued graphs: A variational approach. Ann Appl Stat. 2010;4: 715–742. doi:10.1214/10-AOAS361

5. Hubert L, Arabie P. Comparing partitions. J Classif. 1985;2: 193–218. doi:10.1007/BF01908075

6. Brose U. Complex food webs prevent competitive exclusion among producer species. Proc R Soc Lond B Biol Sci. 2008;275: 2507–2514. doi:10.1098/rspb.2008.0718

7. Brose U, Williams RJ, Martinez ND. Allometric scaling enhances stability in complex food webs. Ecol Lett. 2006;9: 1228–1236. doi:10.1111/j.1461-0248.2006.00978.x

8. Yodzis P, Innes S. Body-size and consumer-resource dynamics. Am Nat. 1992;139: 1151–1173.

9. Otto SB, Rall BC, Brose U. Allometric degree distributions facilitate food-web stability. Nature. 2007;450: 1226–1229. doi:10.1038/nature06359

10. Brose U, Berlow EL, Martinez ND. Scaling up keystone effects from simple to complex ecological networks. Ecol Lett. 2005;8: 1317–1325. doi:10.1111/j.1461-0248.2005.00838.x

11. Kéfi S, Berlow EL, Wieters EA, Joppa LN, Wood SA, Brose U, et al. Network structure beyond food webs: mapping non-trophic and trophic interactions on Chilean rocky shores. Ecology. 2015;96: 291–303. doi:10.1890/13-1424.1
